# Unravelling genome-wide mosaic microsatellite mutations at single-cell resolution

**DOI:** 10.64898/2026.02.04.703915

**Published:** 2026-02-06

**Authors:** Chunyi Wang, Wenxuan Fan, Weixiang Wang, Yonghe Xia, Jinhong Lu, Xiaoyu Ma, Jichuan Yu, Yunchao Zheng, Yan Luo, Wenlong Li, Qing Yang, Meizhen Lin, Huan Liu, Yangning Lan, Chengyu Li, Xiaodong Liu, Danyang He, Shang Cai, Xiuyan Yu, Dan Zhou, Manolis Kellis, Xushen Xiong, Qi Xie, Yanmei Dou

**Affiliations:** 1. School of Life Sciences, Westlake University, Hangzhou, Zhejiang, 310024, China; 2. Westlake Laboratory of Life Sciences and Biomedicine, Hangzhou, Zhejiang, 310024, China; 3. College of Life Sciences, Zhejiang University, Hangzhou, Zhejiang, China; 4. Fudan University, Shanghai, 200433, China; 5. Liangzhu Laboratory, Zhejiang University, Hangzhou, Zhejiang, China; 6. Department of Breast Surgery, Second Affiliated Hospital, Zhejiang University, Hangzhou, Zhejiang, China; 7. The Second Affiliated Hospital, School of Public Health, Zhejiang University School of Medicine, Hangzhou, China; 8. Computer Science and Artificial Intelligence Laboratory, Massachusetts Institute of Technology, Cambridge, MA 02139, USA; 9. These authors contributed equally

## Abstract

Short tandem repeats (STRs), or microsatellites, are highly mutable genomic elements that modulate gene regulations and are implicated in a range of human diseases. However, detecting mosaic STR mutations at single-cell resolution remains challenging due to both technical and biological complexities. To address this, we developed BayesMonSTR, a robust algorithm that enables accurate detection of mosaic STR mutations. Using this tool in single-cell analysis of human tissues, we reveal an accumulation of longer mosaic STR insertions and deletions (indels) in aging mitotic and post-mitotic cells. Strikingly, prefrontal cortex (PFC) neurons accumulate a higher burden of STR mutations than B cells or lung epithelium, with aged neurons exhibiting a particularly pronounced increase in longer STR deletions. These mutations are enriched at transcription start sites (TSSs) and active enhancers of highly expressed genes. Our work establishes a foundation for genome-wide, hypothesis-free discovery of disease-associated mosaic STR mutations and reveals a previously unexplored landscape of mosaic STR variation in development and aging.

## Introduction:

Somatic mosaicism—postzygotic mutations acquired over a lifetime—reflects the cumulative interplay between genome instability, environmental exposures, and cellular repair processes. While mosaicism contributes to aging and diseases^1^, its detection has focused predominantly on SNVs^2,3^ and structural variants^4–6^, overlooking STRs. STRs, spanning ~1-5% of the human genome, exhibit mutation rates up to 1,000-fold higher than non-repetitive regions due to polymerase slippage and replication errors^7^. Importantly, STRs regulate transcription, facilitate chromatin interactions, and are linked to nearly a hundred human disorders—a large proportion of which are neurological^8,9^. However, technical challenges—such as PCR artifacts, alignment ambiguities^7^, and the inability to resolve STRs at single-cell resolution^10^—have obscured the landscape of mosaic STR mutations in aging tissues. Existing STR genotypers^11–13^ are designed for germline variants or bulk tissues^14^, failing to distinguish mosaic mutations from artifacts in single-cell data^15^. This gap has limited our ability to explore hypotheses about age-related STR genome instability and their roles in aging and disease.

Recognizing the critical gap in current methodologies, we developed BayesMonSTR, a computational framework that integrates probabilistic read segmentation, haplotype phasing, and a hierarchical Bayesian model to detect mosaic STR mutations with nucleotide-level resolution. By integrating comprehensive layers of data and quantifies all aspects in terms of probability, BayesMonSTR resolves ambiguities in extracting imperfect STR alleles (e.g., interruptions, truncated motifs) from noisy sequencing data, while rigorously filtering out technical artifacts and germline polymorphisms. Using this tool, we revealed the profiles of mosaic STR mutations in both post-mitotic and mitotic tissues during aging. Notably, neurons from healthy individuals accumulate age-associated long STR deletions, which are specifically enriched in the regulatory regions of highly expressed genes. Analysis of snATAC-seq data from the PFC neurons of Alzheimer’s disease (AD) patients reveals a convergence of mosaic STR mutations in active regulatory regions of AD risk genes.

Our approach maps unexplored landscapes of mosaic STR mutations across aging tissues, enabling the genome-wide, hypothesis-free discovery of novel disease-associated variants. BayesMonSTR is open source, licensed under the MIT License, and publicly accessible on GitHub at https://github.com/douymLab/BayesMonSTR.

## Results:

### Overview of BayesMonSTR

BayesMonSTR is a computational framework designed for the robust detection of mosaic STR mutations from single cell sequencing data. The input to BayesMonSTR consists of sequencing reads and an STR panel, which defines a set of STR loci on the reference genome. For this study, we utilized a commonly adopted STR panel containing ~1.6 million loci^11^, ensuring a relatively comprehensive coverage of the genome’s repetitive regions. Notably, unlike most existing methods that primarily estimate STR mutations length changes^16,17^, BayesMonSTR provides nucleotide-level resolution, enabling not only the inference of STR length changes but also the identification of mosaic point mutations within the STR and its flanking regions. This capability significantly enhances the resolution and biological interpretability of STR mutation detection.

**Fig. 1:**
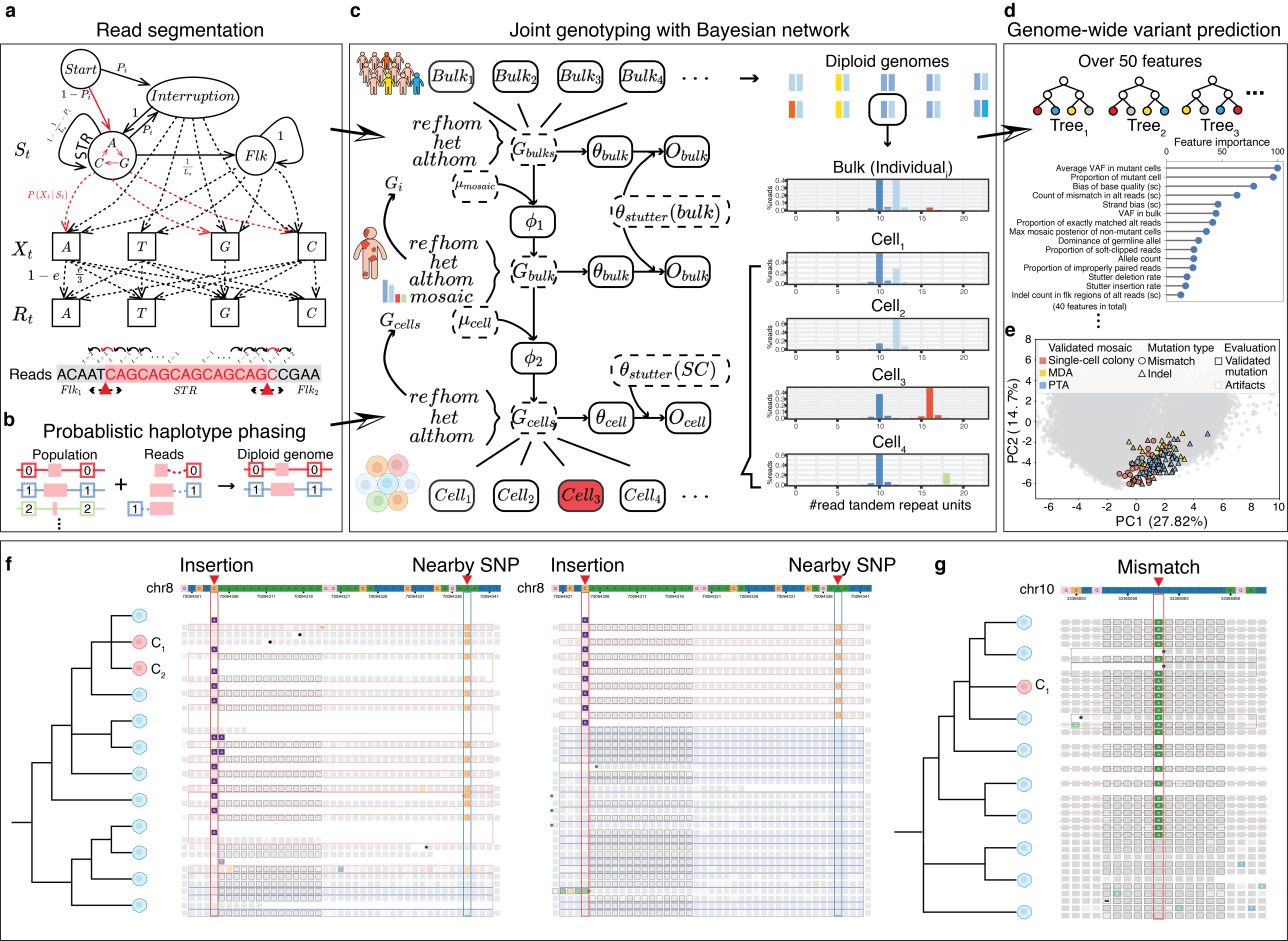
Overview of BayesMonSTR. (**a**) BayesMonSTR employs an HMM to probabilistically segment each sequencing read, distinguishing the STR region from its flanking sequences. (**b**) STR alleles are assigned to their chromosomal haplotypes by statistically linking them to the alleles of neighboring heterozygous variants. (**c**) A Hierarchical Bayesian Network integrates three layers of data—population-level sequencing, individual-level bulk sequencing, and single cell sequencing—to calculate individual- and cell-level genotype posteriors. (**d**) An RF model, trained on over 50 carefully chosen read-level and genome-level features, classifies true mosaic mutations from artifacts. (**e**) PCA constructed from top 40 features shows that validated mosaic variants from independent single-cell sequencing platforms cluster in similar regions, highlighting the robustness of BayesMonSTR. (**f**) Illustration of a mosaic 1-bp insertion. A 1-bp insertion mutation detected in single-cell colony data is shown. Supporting sequencing reads covering the variant site are displayed for two distinct cells harboring the mutation. (**g**) A cell-specific base substitution within a short tandem repeat (STR) region, identified from single-cell whole-genome sequencing (scWGS) data.

To process the sequencing reads, we employed a probabilistic hidden Markov model (HMM) for read segmentation, separating each read into STR and flanking regions (Fig. 1a, Methods). This read segmentation step ensures precise delineation of repetitive sequences from adjacent regions, reducing potential biases in downstream analyses, and the segmented reads were then used to generate sequence-based STR alleles (Supplementary Fig. 1).

The segmented reads were then used to generate haplotype-resolved STR alleles, a crucial step for distinguishing true STR mutations from sequencing artifacts. To enhance the accuracy of allele assignment, we implemented a probabilistic phasing framework (Fig. 1b, Methods). This framework integrates population haplotype information with sequencing reads from individual samples, enabling the calculation of linkage probabilities between STR alleles and nearby germline heterozygous single nucleotide polymorphisms (hSNPs).

BayesMonSTR then employs a hierarchical Bayesian network to jointly infer germline and somatic mosaic genotype posteriors integrating multiple layers of data, including population allele frequencies, bulk sequencing data and single cell sequencing data (Fig. 1c, Methods). After calculating the somatic posteriors for individual single cells, we applied a dynamic programming approach to estimate the probability that a given site contains at least one somatic cell within an individual (Methods). The key parameters of the Bayesian network were estimated using specialized methods based on real-world data. For example, stutter-error parameters were derived from a length-based stutter error model (Supplementary Fig. 2, Methods), while imbalanced allele frequencies in whole-genome amplified single-cell sequencing data were modeled using a piece-wised Gaussian process regression approach (Methods).

Following Bayesian genotype inference, over 50 carefully chosen features were extracted to distinguish mosaic mutations from artifacts (Fig. 1d, Methods). These features include metrics capturing read and base qualities, allele imbalances, and STR-specific error patterns, *etc.* (Supplementary Table 1). By generating a meticulously curated training set of over 10,000 orthogonally evaluated and manually checked candidate STR mutations from three different single-cell sequencing platforms (Supplementary Table 2), we employed a Random Forest (RF) classification model to identify mosaic STR mutations across the genome (Fig. 1e, Methods). Notably, we trained two separate models: one for phasable sites with at least one nearby hSNP and another for nonphasable sites, which we refer to as ‘Phasable mode’ and ‘Nonphasable mode’. In the RF model for Phasable mode, haplotype-phasing-related features were incorporated (Methods).

The comprehensive framework enables BayesMonSTR to achieve accurate detection of base pair changes within STR regions, including both indels and base substitutions. Figures 1f and 1g illustrate examples of mosaic STRs detected from two public datasets derived from two different sequencing platforms^18,19^. These visualizations, generated with our newly developed software BayesMonSTR-INSIGHT^20^ specifically designed to provide advanced visualization of mosaic mutations, highlight BayesMonSTR’s capability to accurately identify both indels (Fig. 1f) and mismatches (Fig. 1g).

### Accurate detection of mosaic STR indels and mismatches from single cell sequencing data

**Fig. 2:**
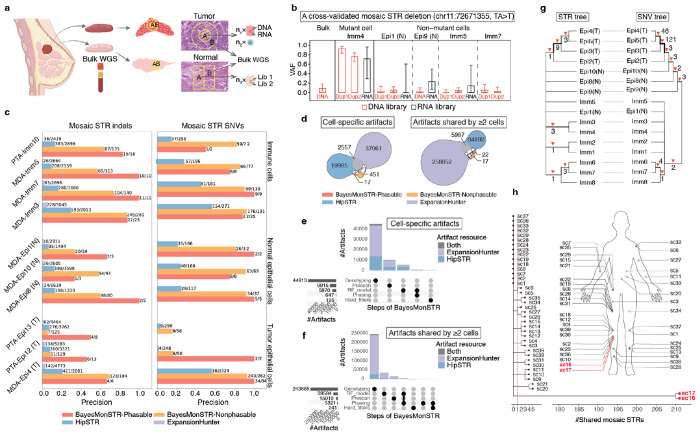
Accurate detection of mosaic STR indels and SNVs from single cell sequencing data. (**a**) To construct a gold-standard benchmarking dataset, we used 28 single cells from a breast tissue sample, comprising six epithelial cells (Epi) from tumor tissue (T), 12 epithelial cells from normal tissue (N), and 10 immune (Imm) cells. Of these, 12 cells had duplicate libraries and were sequenced twice, while 15 cells underwent both DNA and RNA sequencing from the same single cell. Single cell DNA was amplified using two widely adopted strategies, PTA and MDA. (**b)** An illustrative site validated through both duplicate library sequencing, and RNA-seq derived from the same single cell. The mutant reads were also present in the bulk DNA-seq from the adjacent tissue. Error bars represent 95% confidence intervals of the mutant-cell VAFs calculated using binomial sampling. (**c**) BayesMonSTR achieved a multi-fold improvement in detecting cell-specific mosaic STR mutations, compared to the adapted versions of HipSTR and ExpansionHunter. (**d-f**) For both cell-specific and shared variants, BayesMonSTR demonstrated a significant improvement in accuracy, largely due to its comprehensive framework, which includes effective probabilistic read segmentation, haplotype phasing, joint genotyping, and the incorporation of informative features into the RF classification model. In panels **e-f,** solid points represent artifact mutations called by other tools that were filtered out by BayesMonSTR’s genotyping, RF prediction model or hard filters. (**g**) The phylogenetic tree reconstructed using mosaic STR mutations resembles the tree built from point mutations, while providing enhanced resolution in certain branches. The width of the branch was positively correlated with the number of mutations that arise at the branching point. Numbers indicate the specific number of mutations that arose at each branching point. (**h**) From a public single cell colony dataset which includes two clones from adjacent skin samples as well as samples with multiple clones from remote tissues, BayesMonSTR detected the highest number of shared STR mutations in the two clones from the adjacent skin samples. The human figure was adapted from the image available at https://scv.cancervision.com/Screen1/DB10.

Single cells possess only a single nucleus, which is used for sequencing, and mosaic mutations are typically present in a small fraction of cells or are cell specific. This makes it extremely challenging to assess the validity of mosaic mutations detected from real-world single cell sequencing data. To address this challenge, we constructed a ‘gold-standard’ benchmarking dataset, providing a more controlled and reliable approach for validation (Fig. 2a).

For this, we sequenced 28 single cells from a breast tissue sample, comprising six epithelial cells from the breast tumor tissue (Lin−EpCAM+CD49f+), 12 epithelial cells from the normal breast tissue (Lin−EpCAM+CD49f+), and ten Lin+ immune cells (Supplementary Fig. 3, Methods). Among these, nine cells were processed as duplicate libraries and sequenced twice, while 14 cells were subjected to both DNA and RNA sequencing from the same single cells. Single-cell DNA was amplified using two widely adopted strategies: MDA^21^ and PTA^22^ (Methods). Additionally, adjacent tissues were sequenced using bulk whole-genome sequencing (WGS). This comprehensive approach allowed us to generate three distinct types of orthogonal benchmarking data: (1) duplicate cell libraries, (2) single-cell DNA and RNA sequencing, and (3) bulk WGS sequencing from adjacent tissues. Figure 2B illustrates a validated site using the three orthogonal approaches.

The performance of BayesMonSTR was evaluated against HipSTR^11^ (version 0.6.2) and ExpanshionHunter^12^ (version 5.0.0). Notably, we adapted the state-of-the-art germline STR callers for single cell sequencing data. By applying a series of hard filters (Methods), we enabled these tools to detect mosaic STR mutations.

Figures 2c–g presents the results generated using the gold-standard benchmarking dataset described above. Across these tests, we demonstrate that BayesMonSTR achieves a remarkable multi-fold improvement in precision for detecting mosaic STR mutations (Fig. 2c, Supplementary Fig. 4a–b, Supplementary Table 3-4). Specifically, BayesMonSTR accurately identifies both cell-specific and shared STR mutations in both tumor-tissue cells and normal-tissue cells, encompassing both indels and base substitutions. One notable observation is that the accuracy of mosaic detection from PTA data was generally lower, likely due to the 9-10 rounds of PCR involved in the PTA library preparation stage (Methods), which introduced additional DNA-slippage errors (Supplementary Fig. 2).

To better understand the superior performance of BayesMonSTR compared to other software tools, we thoroughly examined artifact variants generated by different tools. Our analysis (Fig. 2d–f, Supplementary Fig. 4c–g) revealed that BayesMonSTR achieved a significant improvement in accuracy due to its comprehensive framework, which includes the joint genotyping with stutter-error models, effective probabilistic haplotype phasing, and a series of informative features integrated into the RF classification model.

As mentioned above, the hypermutable nature of STR regions makes them promising targets for *in vivo* lineage tracing. By leveraging predicted mosaic STR mutations shared across multiple cells, we constructed a lineage tree^23^ (Supplementary Table 5, Methods). The tree generated from STR mutations across ~1% of the genome resembles the topology of the tree constructed from SNVs detected in the remaining 99% of the genome (Fig. 2g, Methods), supporting the potential using STR mutations for cost-efficient lineage tracing.

To further evaluate the robustness of BayesMonSTR, we analyzed three publicly available single-cell sequencing datasets generated using diverse strategies, including single-cell whole genome amplification (WGA) and single-cell colonies^18,24–26^. These datasets were specifically chosen based on the availability of orthogonal data for cross validation, such as duplicate single-cell libraries or adjacent tissue samples. Across multiple benchmarks, BayesMonSTR consistently achieved a superior accuracy for detecting mosaic STR mutations, underscoring its robustness (Supplementary Fig. 4h). For example, in the single-cell colony dataset comprising cells from adjacent skin samples, BayesMonSTR identified the highest number of shared variants between the cells from adjacent skin samples, compared to remote tissues, further validating its performance (Fig. 2h).

Finally, we evaluated BayesMonSTR on simulated data with spike-in mutations (Supplementary Fig. 5, Supplementary Table 6, Methods). This analysis demonstrated that BayesMonSTR achieved substantially higher precision than other tools by effectively excluding most artifacts and germline variants, while maintaining a competitive sensitivity profile (0.4-0.6 vs. 0.6-0.8).

### Landscape of mosaic STRs from mitotic and post-mitotic cells

Genome instability arises from a complex interplay of biological and chemical processes, including replication stress, DNA repair activity, transcription-associated DNA damage and repair, endogenous metabolic byproducts, exogenous environmental factors, *etc.* To investigate STR-associated instability in different contexts, we analyzed publicly available datasets from three distinct cell types with varying proliferative potentials and environmental exposures: (1) B cells^27^ from the blood, which undergo frequent replication during their lifespan ; (2) lung epithelial cells^28^, which are proliferative particularly during the wound-healing process, and (3) neurons^26^, which cease dividing at an early developmental stage and are highly transcriptionally active.

From the collected datasets, BayesMonSTR identified 4,008 mosaic STR indels and 971 mosaic STR SNVs (defined as SNVs within or flanking STRs by ±5 bp) from 56 B cells across 14 individuals. Additionally, it identified 1,745 mosaic STR indels and 3,447 STR SNVs from 160 lung epithelial single-cell colonies across 16 individuals, and 13,488 mosaic STR indels and 2,353 STR SNVs from 126 neurons across 19 individuals (Supplementary Table 7). The mutation rate per cell per base pair ranges from approximately 10^−6^ to 10^−5^, significantly higher than the reported point mutation rate outside of microsatellite regions^2^. The mutation signatures differ across tissues (Methods, Supplementary Fig. 6), and the overall exonic indel mutation rate was about 60% lower than expected (p = 1.08×10^−121^, two-tailed binomial test, Table 1, Supplementary Table 8), suggesting a strong purifying selection.

### Age-related accumulation of longer STR indels

**Fig. 3:**
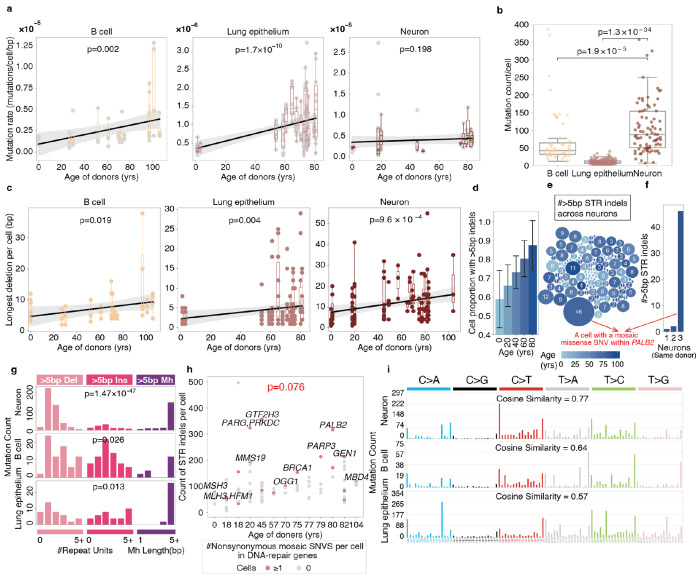
Mosaic STR mutations by age across tissues and mutation mechanism analysis. (**a**) Mosaic mutations within and flank STR regions, including both indels and point mutations, accumulate with age in B cells and lung epithelial cells. In contrast, the mutation rate remains relatively stable in non-dividing neurons across donors of different ages. Linear regression equations were derived using the least squares method. P-values were calculated using a one-tailed t-test to test whether the regression coefficient is significantly greater than zero. The shaded areas around each curve represent the 95% CIs, assuming the differences between predicted values and observed values follow a t-distribution. Due to higher stutter errors in PTA-amplified cells, 1-bp STR indels from PTA cells showed relatively low validation rates, so only MDA-amplified neurons were used in panel a. Longer STR indels, which have higher signal-to-noise ratios, were included in the analysis of panels c-j. All long indels passed manual validation. (**b**) The total mosaic mutation burden per cell in neurons was significantly higher compared to the other two cell types. P-values were calculated using a two-tailed Mann-Whitney U test. (**c**) Across all three cell types, the length of longest mosaic STR contraction per cell increased significantly by age. The regressions, p-values, and CIs were calculated using the same approach as in panel A. (**d**) Estimated proportion of neurons carrying mosaic STR indels greater than 5bp by age. The proportion of neurons was predicted by a linear regression. Error bars indicate the 95% CIs assuming the differences between predicted values and observed values follow a t-distribution. P-values were calculated with a two-tailed Mann-Whitney U test. All insertions greater than 5 bp included in panels d-g have passed the manual verification process. (**e**) Mosaic STR indels greater than 5 bp were prevalent across neurons, particularly in aged neurons. Each circles represents one individual neuron, with the numbers inside each circle indicating the count of >5bp STR indels acquired by each neuron. The gradient color scale represents the varying ages of the donors. The neuron carrying 46 >5 bp STR indels also harbors a missense mutation in the DNA repair gene *PLAB2*. (**f**) Distribution of >5 bp STR indels across three neurons from the same individual. The cell (same with the cell in panel e that carries 46 long STR indels) with the *PLAB2* missense mutation exhibits a substantially higher mutational burden of >5bp STR indels than the other two neurons. (**g**) Mutation signatures of >5bp STR indels across three cell types. Mh refers to microhomology. (**h**) The presence of nonsynonymous mosaic SNVs in DNA repair genes was marginally significantly associated with an increased count of mosaic STR indels in neurons. Each point represents one cell, and red points indicate cells carrying at least one nonsynonymous mosaic SNV in DNA-repair related genes. See Methods for details. Analyses were performed using GLMM with a negative binomial distribution, including a random intercept for individual to account for within-individual correlations. Critical genes associated with DNA repair were annotated beside the mutant cells. (**i**) Three-nucleotide context mutation signature profile of STR-region SNVs across three cell types. The numbers in the figure represent the cosine similarity between each mutation profile and the established patterns of genome-wide mosaic SNVs in prefrontal cortex (PMID 41166474).

We observed that the total burden of mosaic mutations within and flanking STR regions—including both indels and point mutations—increases with age in B cells (p=0.002, one-tailed t test) and lung epithelial cells (p=1.7 × 10^−10^, one-tailed t test). In contrast, mutation burden remains relatively stable in post-mitotic neurons across donors of different ages (p=0.198, one-tailed t test, Fig. 3a). This is consistent with the established understanding that STR-associated mutations arise primarily from replication slippage during DNA proliferation. Notably, neurons exhibited a significantly higher mosaic mutation burden than the other two cell types (vs. B cells: p = 1.3 × 10^−34^; vs. lung epithelium: p = 1.9 × 10^−5^; two-tailed Mann–Whitney U test; Fig. 3b), suggesting that mosaic STR mutations in neurons accumulate rapidly during early development.

Indels of different lengths arise from distinct mutational mechanisms. Longer indels affect more genomic sequence, therefore posing a higher risk of DNA structure disruption. To determine whether cells in healthy individuals accumulate longer STR indels with age, we analyzed indel length distributions across three cell types. Interestingly, both indel length (Fig. 3c) and the burden of long deletions (Supplementary Fig. 7a) increased significantly with age in lung epithelium, B cells, and neurons. Specific p-values for the increase in indel length were: lung epithelium (p=0.004), B cells (p=0.019), and neurons (p=9.6 × 10^−4^; one-tailed t-test); p-values for the increased long-deletion mutation rates were: lung epithelium (p=0.023), B cells (p=0.091), and neurons (p=8.7 × 10^−4^; one-tailed t-test). Full mutation list is provided in Supplementary Table 9. This trend was particularly striking in neurons, as their burden of 1-bp STR mutations shows no age-related increase (p=0.456, one-tailed t test, Supplementary Fig. 7b), suggesting that longer STR indels are generated via distinct, age-dependent mechanisms, which shape a unique mutational spectrum over the neuronal lifespan.

These longer STR indels are prevalent across neurons. By the age of 80, approximately 85% of neurons from healthy individuals carry at least one STR indel greater than 5 bp (Fig. 3d, Methods). Across the 126 neurons from 19 individuals, while most cells harbor less than ten such STR indels, some cells contain more (Fig. 3e).

Notably, one aged neuron from an 80-year-old female exhibited nearly 50 long STR indels. Intriguingly, this neuron—which shows a high burden of long STR indels—carries a nonsynonymous mosaic exonic SNV in *PALB2* (Partner and Localizer of BRCA2, Supplementary Table 10, Methods), an essential gene involved in DNA repair^29^. This cell harbored a >30-fold higher number of >5bp mosaic STR indels than other two neurons from the same individual (Fig. 3f), suggesting a possible defect in microsatellite repair driven by mosaic SNVs in DNA repair–related genes.

### Intrinsic microsatellite mutagenesis in aging tissues

To investigate the mechanisms underlying longer STR indels, we analyzed the properties of the microsatellites in which they occur, focusing on tract length, motif composition, and genomic location. This analysis revealed that longer indels preferentially occur in STRs that are themselves longer (p=1.9×10^−21^, two-tailed Mann-Whitney U test, Supplementary Fig. 8a) and have higher GC content (p=1.0×10^−32^, two-tailed Kolmogorov-Smirnov test, Supplementary Fig. 8b). We further conducted mutation signature analyses (Methods). The indel signatures of >5bp STR indels across all three cell types resembled those observed in *MSH2* (MutS homolog 2) and *MSH3* (MutS homolog 3) gene knockouts^30^ (Supplementary Fig. 8c), implicating potential deficiencies in human MutSβ, which normally binds unpaired DNA loops commonly found in STR regions^31^.

Notably, while >5 bp insertions and deletions occurred at similar frequencies in B cells and lung epithelium, neurons displayed a significant bias toward deletions over insertions (p=1.47×10^−47^, one-tailed Binomial test, Fig. 3f). Among these deletions, 29.8% were flanked by ≥5 bp microhomology—a hallmark of microhomology-mediated end joining (MMEJ), which typically generates long deletions. This pattern suggests increased double-strand break formation and preferential MMEJ repair in aging neurons^32^.

To further investigate whether STR indel burden correlates with DNA repair capacity, we examined the relationship between STR indel counts and the presence of nonsynonymous exonic mosaic SNVs in DNA repair genes across neurons (Supplementary Table 10). Using a negative binomial generalized linear mixed model (GLMM) with individual as a random effect to account for donor-level variability, we tested for a positive correlation between per-cell STR indel burden and the presence of such mutations. Neurons carrying nonsynonymous SNVs in DNA repair genes showed a marginally significant increase in STR indel burden (p = 0.076, negative binomial GLMM, Fig. 3g, Methods). Intriguingly, among the neurons from each donor, several exhibited substantially higher STR indel counts than the others from the same individual. Notably, these outliers harbored nonsynonymous mutations in key DNA repair genes, mainly including several double strand break repair genes (*PRKDC, PARP3, BRCA1, PALB2, GEN1*)^33^ and *GTF2H3*, a core factor in nucleotide excision repair (NER)^34^. Together, these results further support the link between neuronal STR indels and age-related DNA repair deficiencies driven by genomic instability.

Beyond indels, we further examined the signatures of mosaic SNVs within STR regions. Notably, STR-region SNVs largely mirrored the established patterns of extra-STR mosaic SNVs—attributed mainly to oxidative damage and cytosine deamination^35^ (Fig. 3i, Supplementary Fig. 8d). Together with our findings on indel signatures and repair-pathway involvement, these results paint a complex picture of microsatellite mutagenesis in aging, marked by both age-associated genomic instability and the engagement of specific DNA repair processes.

### Mosaic STR indels in neuronal open chromatin

**Fig. 4:**
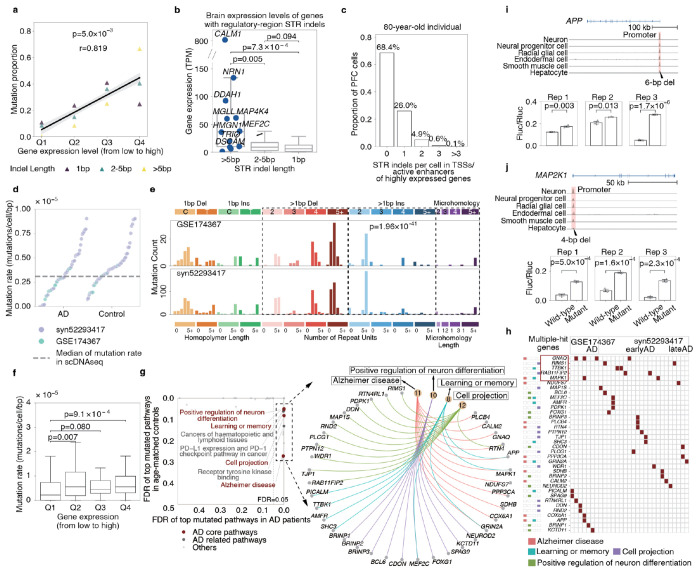
Regulatory mosaic STR indels in neurons. (**a**) Mosaic STR indels in neurons are enriched in TSSs and active enhancers of highly expressed genes. The number of variants is indicated beside each data point. Q1-Q4 represent expression level quantiles from low to high (Methods), and the brain gene expression data were obtained from ENCODE. Linear regression equations were derived using the least squares method. P-values were calculated using a one-tailed t-test to test whether the regression coefficient is significantly greater than zero. The shaded areas around each curve represent the 95% CIs, assuming the differences between predicted values and observed values follow a t-distribution. (**b**) Genes with >5 bp mosaic STR indels in the TSS regions or active enhancers exhibit elevated gene expression levels, compared to genes with shorter indels in the TSS regions or active enhancers. P-value was calculated using a two-tailed Mann-Whitney U test. (**c**) Based on Poisson modeling—using the product of the total length of regulatory regions and the median mutation rate as the parameter μ—we estimate that over 30% of neurons in an 80-year-old individual carry at least one mosaic STR indel within the TSSs or active enhancers of highly expressed genes. (**d**) snATAC-seq samples exhibit mutation rates highly consistent with those observed in scWGS data. (**e**) A significant excess of >1 bp deletions over >1 bp insertions was observed in snATAC-seq data. P-value was calculated using a one-tailed Binomial test. (**f**) The STR mutation rate in active regulatory regions is increased in more highly expressed genes. P-values were calculated with a two-tailed t-test. (**g**) In AD patients, but not in age-matched healthy controls, STR indels within transcription start sites (TSSs) and active enhancers were significantly enriched for genes in AD-related pathways (Methods). (**h**) Multiple key genes in AD pathways contained multi-hit STR indels in their active regulatory regions across different patients. “earlyAD” and “lateAD” indicate early-stage AD patients and late-stage AD patients. (**i-j**) Functional validation of somatic STR indels in regulatory regions using luciferase assays. Normalized levels of H3K4me3 and H3K27ac—key histone modifications linked to promoter and enhancer activities—were obtained from the ENCODE project. Pink regions indicate the promoter or enhancer regions of the gene, with indels highlighted by black triangles. Luciferase assays in HEK293T cells revealed that a 6-bp deletion in the TSS of *APP* and a 4-bp deletion in the TSS of *MAP2K1* significantly enhanced gene expression (P < 0.05, two-tailed t-test), with error bars representing standard deviation (3 biological replicates).

Given the established involvement of microsatellites in gene regulation^36^, we investigated the distribution of STR indels in active regulatory regions across neurons. We found these mutations are more enriched in the TSS and active enhancers of genes with high expression levels in the brain (p=5.0×10^−3^, one-tailed t-test, Fig. 4a). Notably, longer STR indels showed the strongest enrichment (Fig. 4b). Given the mutation prevalence, over 30% of neurons in an 80-year-old individual would harbor at least one mosaic STR indels within TSSs of active enhancers of highly expressed genes based on Poisson sampling (Fig. 4c).

To further explore the enrichment of STR mutations in neuronal open chromatin regions, we next adapted the BayesMonSTR framework to detect mosaic STR indels directly from snATAC-seq data (Methods). Based on snATAC-seq data from the prefrontal cortex (PFC) of 55 AD patients and 55 age-matched healthy controls from two independent datasets^37,38^, the mutation rate closely matched that observed in sc-WGS data (Fig. 4d, Methods). Like patterns in neuronal scWGS data, we observed a significant excess of long deletions over long insertions (p=1.96×10^−41^, one-tailed Binomial test, Fig. 4e). Crucially, the STR mutation rate was elevated in the active regulatory regions of highly expressed genes, mirroring the pattern we observed in scWGS data (Fig. 4f).

Intriguingly, while the per-cell mutation rate was similar between AD patients and controls (Fig. 4d), STR indels in AD patients located within TSSs and active enhancers were significantly enriched in genes associated with AD-related pathways (Fig. 4g–h, Methods). These included ontologies and genes related to learning and memory, amyloid-β (Aβ) pathway and synaptic function, positive regulation of neuron differentiation, cell projection, etc. (Supplementary Table 11). Many of the affected genes are well-recognized AD risk genes, such *APP* (amyloid beta precursor protein)^39^, *CD2AP* (CD2-associated protein)^40^, *PICALM* (phosphatidylinositol binding clathrin assembly protein)^41^, *MAPK1* (mitogen-activated protein kinase 1)^42^, *MEF2C* (Myocyte-specific enhancer factor 2C)^43^, etc.

To test the function of these mutations, we further conducted luciferase assays of six STR indels within the TSS regions and active enhancers of *APP, CD2AP, MAP2K1, PLCB4, BRINP*, and *PPP3A* (Methods). Remarkably, experimental results showed that 4 out of 6 mutations led to significantly disrupted gene expressions in 3 replicated experiments in 293T cells (Fig. 4i–j, Supplementary Fig. 9). These findings indicate that microsatellites in active regulatory regions of highly brain-expressed genes are particularly mutation-prone and may affect gene expression.

## Discussion

Short tandem repeats are vastly overrepresented in the human genome, occurring far more frequently than what would be expected by chance^44^. Serving as valuable genomic elements for fast evolving, the hypermutable nature of STRs also makes them vulnerable to harmful mutations. The single-cell genomic landscape of mosaic STR mutations has not been explored before due to technical difficulties and the complexity of these sequences. In this study, we present BayesMonSTR, the first tool to accurately detect genome-wide somatic STR mutations in single cells. This capability provides a unique opportunity to map the unexplored dynamic landscape of STR mutagenesis and enables the hypothesis-free discovery of mosaic STR mutations associated with aging and disease.

By characterizing mosaic STR mutations across mitotic and post-mitotic tissues, we uncovered heterogeneous mutation rates and profiles. Unexpectedly, the mutation rate in post-mitotic neurons was significantly higher than in mitotic B cells and lung epithelial cells from age-matched individuals. The accumulation of longer STR indels in neurons with aging was especially intriguing. Notably, we observed a neuron-specific increase in longer STR deletions versus insertions over time, a pattern distinct from that seen in mitotic B cells and lung epithelium. Mechanistic analysis further suggested that these mutations may arise from double-strand DNA breaks and coupled MMEJ. Furthermore, we found that longer STR indels accumulating in neurons with age were enriched in the active regulatory regions of highly expressed genes; In published snATAC-seq datasets from AD patients PFC cells, these mosaic STR indels were significantly enriched in established AD risk genes and associated pathways.

Finally, BayesMonSTR provides a versatile, unified toolkit for detecting mosaic STR mutations from single-cell WGS, snATAC-seq (BayesMonSTR-ATAC), and bulk WGS data (BayesMonSTR-BulkMonSTR)^45^, as well as enhanced visualization of STR mutations (BayesMonSTR-INSIGHT)^20^. By enabling precise characterization across these platforms, it opens new opportunities to investigate the contribution of STR mutations to aging and disease.

## Methods

### Sequence alignment and data preprocessing

Raw FASTQ reads were aligned to the GRCh37 reference genome (human_g1k_v37_decoy) using BWA-MEM (v0.7.18). Duplicate reads were marked with Picard MarkDuplicates (v2.27), and base quality score recalibration (BQSR) was performed with the Genome Analysis Toolkit (GATK v4.2). Germline variants were called with GATK HaplotypeCaller on both MDA single-cell and matched bulk data and filtered using Variant Quality Score Recalibration (VQSR) following GATK Best Practices. Common germline heterozygous SNPs (gnomAD^1^ allele frequency >0.1%) were selected for downstream haplotype phasing with Eagle2 (v2.4.1)^2^.

### Stutter-error estimation

To reduce amplification- and sequencing-related artifacts introduced by *in vitro* polymerase slippage, BayesMonSTR models STR stutter errors using a geometric-distribution^3^. The stutter error probability *p*(*r_m,c_*|*a* = *j*) for *m*-th read *r* for cell *c* in the given allele *j* was modeled as follows:

(1) $$P\left(r_{m,c}|a=j\right)=\{\begin{array}{ll} 1-u-d, & r_{m,c}=r_{a}\\ u\rho {\left(1-\rho \right)}^{r_{m,c}-r_{a}-1}, & r_{m,c}>r_{a}\\ d\rho {\left(1-\rho \right)}^{r_{a}-r_{m,c}-1}, & r_{m,c}<r_{a} \end{array}$$

Where:

- *u* denotes the total mutation rate of artifact insertions.
- *d* denotes the total mutation rate of artifact deletions.
- *ρ* denotes the success probability of the geometric model, which belongs to the interval (0,1].

Assuming independence across reads, the genotyping likelihood for each diploid cell is computed using equation 2:

(2) $$P\left(D_{c}|G_{c}=(j,k),\theta_{stutter,cell}\right)=\prod_{m=1}^{n_{reads,c}}\sum_{a\in \{j,k\}}\frac{1}{2}\{\begin{array}{ll} 1-u-d & r_{m,c}=r_{a}\\ u\rho {\left(1-\rho \right)}^{r_{m,c}-r_{a}-1} & r_{m,c}>r_{a}\\ d\rho {\left(1-\rho \right)}^{r_{a}-r_{m,c}-1} & r_{m,c}<r_{a} \end{array}$$

Where:

- *G_c_* denotes the genotype of a given cell.
- *j* and *k* denote the two haplotype alleles.
- *D_c_* denotes the sequencing reads of a given cell *c*.
- The parameter set *θ* denotes the stutter model parameters (*u*, *d*, *ρ*), as described in formular (1).

Of note, reads with mapping quality < 20, as well as reads with mean base quality < 20, were excluded before stutter-parameter estimation.

#### Parameter initialization:

- Given *n* candidate alleles, the initial population frequency for allele *a*, *f_a_*, was set to $\frac{1}{n}$, representing equal prior probability across all alleles.
- The initial mosaic allele fraction parameter μ_msi_ was set to 0.001.
- Stutter error parameters *u*, *d* and *ρ*, which define the geometric model, were initialized to 0.01, 0.01, and 0.9, respectively.

#### E-step:

In the E-step, posterior probabilities of individual germline genotypes were computed using equation 3:

(3) $$P\left(G_{i}^{t}=(j,k)|G_{c}^{t},D_{c},D_{bulk},\theta_{stutter,cell}^{t},\theta_{stutter,bulk}^{t}\right)=\;P\left(G_{i}^{t}=(j,k)\right)\text{*}P\left(D_{bulk}|G_{bulk}^{t}=(j,k),\theta_{stutter,bulk}^{t}\right)\text{*}\prod_{c=1}^{n_{cells,i}}\left[P\left(G_{c}^{t}|G_{i}^{t}\right)\text{*}P\left(D_{c}|G_{c}^{t},\theta_{stutter,cell}^{t}\right)\right]=\;2f_{j}^{t}f_{k}^{t}\text{*}\prod_{m=1}^{n_{reads,i,bulk}}\frac{1}{2}\sum_{a\in j,k}P\left(S_{m,i}=a|\theta_{stutter,bulk}^{t}\right)\text{*}\prod_{c=1}^{n_{cells,i}}\sum \{\begin{array}{c} \frac{1}{2}\mu_{msi}\text{*}\sum_{-k\in A_{i}\backslash \{k\}}\;P\left(D_{c}|G_{c}^{t}=(j,-k),\theta_{stutter,cell}^{t}\right)\\ \left(1-\mu_{msi}\right)\text{*}P\left(D_{c}|G_{c}^{t}=(j,k),\theta_{stutter,cell}^{t}\right)\\ \frac{1}{2}\mu_{msi}\text{*}\sum_{-j\in A_{i}\backslash \{j\}}\;P\left(D_{c}|G_{c}^{t}=(-j,k),\theta_{\mathrm{stutter,cell}}^{t}\right) \end{array}$$

Where:

- *f_j_* denotes population allele frequency for allele *j*.
- *G_i_* denotes the individual germline genotype for individual *i*, and
- *G_c_* denotes the cell genotype.
- *θ* denote the set of stutter error parameters as previously described.
- *D_c_* denotes the sequencing reads of a given cell *c*.

Here we assume *G_i_* is the same as *G_bulk_*, and the prior probabilities of individual genotypes were calculated based on Hardy-Weinberg Theorem. When *j* equals to *k*, the prior probability of a homozygous germline genotype is given by $f_{j}^{2}$.

#### M-step:

We then estimated the error parameters *u*, *d* and *ρ*, and the length-based population allele frequency *f* at *t* + 1-th step for stutter errors in the M-step, and were calculated by equations 4–7, as follows:

(4) $$u^{t+1}=\frac{\sum_{c=1}^{N_{ind}}\;\sum_{m=1}^{N_{reads,c}}\;P\left(S_{m,c,i},r_{m,c,i}|\theta_{stutter}^{t}\right)I\left(r_{m,c,i}>s_{m,c,i}\right)}{N_{reads}}$$

(5) $$d^{t+1}=\frac{\sum_{c=1}^{N_{ind}}\sum_{m=1}^{N_{reads,c}}P\left(S_{m,c,i},r_{m,c,i}|\theta_{stutter}^{t}\right)I\left(r_{m,c,i}<s_{m,c,i}\right)}{N_{reads}}$$

(6) $$\rho^{t+1}=\frac{\sum_{c=1}^{N_{ind}}\sum_{m=1}^{N_{reads,c}}\;P\left(S_{m,c,i},r_{m,c,i}|\theta_{stutter}^{t}\right)I\left(r_{m,c,i}\neq s_{m,c,i}\right)}{\sum_{c=1}^{N_{\mathrm{ind}}}\sum_{m=1}^{N_{reads,c}}\;P\left(S_{m,c,i},r_{m,c,i}|\theta_{stutter}^{t}\right)\left|r_{m,c,i}-s_{m,c,i}\right|}$$

(7) $$f_{j}^{t+1}=\frac{1}{2N_{ind}}\sum_{i=1}^{N_{ind}}\sum_{k=1}^{A_{i}}\left(P\left(G_{i}^{t}=(j,k)|G_{c,i}^{t},D_{c,i},D_{bulk,i},\theta_{stutter}^{t}\right)+P\left(G_{i}^{t}=(k,j)|G_{c,i}^{t},D_{c,i},D_{bulk,i},\theta_{stutter}^{t}\right)\right)$$

Where:

- *N_ind_* denotes total number of individuals.
- The indicator function *I* is used to restrict the computation to reads with insertion, deletion and nonzero stutter for *u*, *d* and *ρ* respectively.
- *p*(*S, r*|*θ*) is read assignment probability for read *r_m,c_* given allele pool *A_i_* (see formula 8).
- *G_i_* denotes the individual germline genotype for individual *i*, and *G_c_* denotes the cell genotype.
- *θ* denote the set of stutter error parameters as previously described.
- *D_c_* denotes the sequencing reads of a given cell *c*.
- *N_reads_* denotes the total number of samples with the same sequencing method. (including bulk, WGA, single cell colonies, etc).
- *N_reads,c_* denotes the total number of reads for sample *c*.
- the indicator function *I* is used to restrict the computation to reads with insertion.
- deletion and nonzero stutter for *u*, *d* and *ρ* respectively.
- *θ* denoted these stutter parameters.
- *f_j_* denotes population allele frequency for allele *j*.

*p*(*S* *r*|*θ*) is read assignment probability for read *r_m,c_* given allele pool *A_i_*:

(8) $$P\left(S_{m,c,i},r_{m,c,i}|\theta_{stutter,cell}^{t}\right)=\sum_{a\in \left\{A_{i}\right\}}P\left(S_{m,c,i}=a\right)\text{*}P(r_{m,c,i}|S_{m,c,i}=a,\theta_{stutter,cell}^{t})=\;=\sum_{a\in \{j,k\}}\sum_{j\in \left\{A_{i}\right\},k\in \left\{A_{i}\right\}}P\left(S_{m,c,i}=a|G_{c,i}^{t}=(j,k)\right)\text{*}\;P\left(G_{c,i}^{t}=(j,k)\right)\text{*}P\left(r_{m,c,i}|S_{m,c,i}=a,\theta_{stutter,cell}^{t}\right)=\;=\sum_{a\in \{j,k\}}\sum_{j\in \left\{A_{i}\right\},k\in \left\{A_{i}\right\}}\sum_{G_{i}^{t}}P\left(S_{m,c,i}=a|G_{c,i}^{t}=(j,k)\right)\text{*}\;P\left(G_{c,i}^{t}=(j,k),G_{i}^{t}|D_{c,i},\theta_{stutter,cell}^{t}\right)*P\left(r_{m,c,i}|S_{m,c,i}=a,\theta_{stutter,cell}^{t}\right)=\;=\sum_{a\in \{j,k\}}\sum_{j\in \left\{A_{i}\right\},k\in \left\{A_{i}\right\}}\sum_{G_{i}^{t}}P\left(S_{m,c,i}=a|G_{c,i}^{t}=(j,k)\right)\text{*}\;P\left(G_{c,i}^{t}=(j,k)|G_{i}^{t}\right)\text{*}P\left(D_{c,i}|G_{c,i}^{t}=(j,k),\theta_{stutter,cell}^{t}\right)\text{*}\;P\left(G_{i}^{t}\right)\text{*}P\left(r_{m,c,i}|S_{m,c,i}=a,\theta_{stutter,cell}^{t}\right)$$

Where:

- *a* is one of the alleles in the alleles pool *A_i_* = {*j, k*} for individual *i*.
- other parameters are defined in Equation (3)

### Probabilistic read segmentation

Accurate detection of repeat regions within sequencing reads is essential for generating candidate STR alleles. Traditional segmentation methods, which rely on alignment coordinates and repeat patterns, often suffer from inaccuracies due to mapping biases and interruptions. These issues lead to fluctuations in the ‘anchor points’ used to distinguish repeat and flanking regions, causing the anchor points to deviate from their expected coordinate across different reads (Extended Data Fig. 1a). To address these issues, we utilized a Hidden Markov Model (HMM) approach to identify repeat regions in sequencing reads.

Notably, our HMM model integrates information from both sequencing reads (including sequencing data and quality scores) and the reference genome (repeat-region and flanking region sequences). It also incorporates prior knowledge of in-frame and out-of-frame interruptions derived from the human reference genome. This design enables robustness to sequence interruptions, sequencing errors, and mutations within STR regions, while integrating base quality scores directly into the segmentation process (Extended Data Fig. 1).

Specifically, the HMMs were designed to model the STR sequences using three hidden blocks (Extended Data Fig. 1b):

- STR block: Represents perfect repeat motifs, capturing the repetitive structure at each STR locus. For example, the motif CAG is modeled with three hidden states—S_C_, S_A_, and S_G_ —within this block.
- Interruption block: Models mismatches and indels within repeat regions. Similar to profile HMM^4^, this block accounts for variations inside the repeat tract.
- Flanking block: Represents sequences flanking the STR. In addition to a general flanking state with equal emission probabilities for A, C, G and T, we introduce a locus-specific 3-bp known flanking states extracted from the human reference genome. These states incorporate sequence context to improve segmentation accuracy.

To initialize the transition probabilities, the initial transition probability from the STR block to the flanking block was defined as 1/L, where L corresponds to the expected segmentation distance before entering the flanking region. For homopolymers, L was set to 5 bp, while for non-homopolymers, L was set to twice the motif length. Furthermore, the initial transition probability from the STR block to the interruption block is estimated through an extensive grid search using a reference panel^3^.

The emission probability matrix integrates both the expected STR motif sequence and base quality scores to improve robustness against sequencing errors. Each hidden state corresponds to an expected nucleotide that receives the highest emission probability. In the flanking block, known 3-bp flanking sequences from the reference genome are assigned predefined emission probabilities, while nucleotides outside this region have equal probabilities across A, C, G, and T. For a given hidden state, if the expected base is *X_t_*, the observed base is *Y_t_*, and the sequencing error rate is e, the emission probability for this base is calculated as follows:

(9) $$P\left(Y_{t}|X_{t}\right)=\{\begin{array}{ll} 1-e, & Y_{t}=X_{t}\\ \frac{e}{3}, & Y_{t}\neq X_{t} \end{array}$$

Where:

- *Y_t_* indicates the observed base in sequencing reads.
- *X_t_* indicates the expected base given a specific hidden state.
- *e* indicates sequencing error rate.

Finally, the boundaries between STR and flanking regions are determined by finding the maximum-likelihood hidden state path using the Viterbi algorithm. Based on this optimal state path, sequencing reads are segmented into STR and flanking segments. In cases of large deletions where the mutant allele length falls below the predefined step length—leading to potential segmentation failure—a BWA-based coordinate mapping approach is used as a backup. Similarly, due to the high sequence complexity at imperfect STR loci with 4-bp, 5-bp, or 6-bp motifs containing interruptions, segmentation performance may be compromised; for such loci, the BWA-based coordinate method is also applied.

### STR-specific read alignment

STR sequences from the segmented reads are used to generate candidate alleles, each comprising the core STR region and 5 bp of flanking sequence on both sides. Segmented reads are subsequently aligned to the candidate alleles using a STR-specific alignment method^3^ to calculate the alignment likelihood. This method accounts for both potential PCR stutter errors and sequencing errors. Specifically, three possible scenarios are considered for the sequencing reads: no stutter error, stutter deletion, and stutter insertion. The calculation of alignment likelihood varies slightly for each scenario, as illustrated in Extended Data Fig. 1c.

Given a haplotype *X* of length *L*, if read *Y* is assumed to be free of stutter errors, the likelihood of the observed alignment is determined by the agreement between each base i in the read and its corresponding base in the haplotype:

(10) $$\text{P}(X,Y)=(1-u-d)\text{*}\prod_{i=1}^{L}Q\left(X_{i},Y_{i}\right)$$

Where:

- *X* denotes the given STR haplotype.
- *Y* denotes the STR haplotype observed in the read.
- *L* denotes the STR length of *X* haplotype.
- *u* denotes stutter insertion rate from the estimated stutter model.
- *d* denotes stutter deletion rate from the estimated stutter model.
- *Q*(*X_i_*, *Y_i_*) represents the agreement score between the *i* -th base of haplotype *X* and the *i*-th base of haplotype *Y*, as defined in Equation (9).

Otherwise, if read *Y* contains a stutter deletion of haplotype *X*—with a deletion size of Δ*L*—we assume that the deletion could occur at any position k within the haplotype, where k ranges from 1 to total_haplotype_length - Δ*L* + 1. The read alignment likelihood is then determined jointly by the probability of stutter deletion and the base-wise agreement between the read and the haplotype. The overall likelihood is obtained by averaging over all possible deletion positions:

(11) $$P(X,Y)=d\rho {\left(1-\rho \right)}^{\frac{\text{Δ}L}{L_{RU}}-1}\text{*}\frac{1}{L-\text{Δ}L+1}\sum_{k=1}^{L-\text{Δ}L+1}\prod_{i=1}^{k-1}Q\left(X_{i},Y_{i}\right)\prod_{i=k+\text{Δ}L}^{L}Q\left(X_{i},Y_{i-\text{Δ}L}\right)$$

Where:

- Δ*L* denotes deletion size in read *Y*.
- *L_RU_* denotes the motif length (in bp).
- *ρ* denotes the success probability of the geometric model, which belongs to the interval (0,1].
- *k* denotes the deletion position in the haplotype *X*.
- Other parameters are defined in Equation (10).

In the third scenario, where the read *Y* results from a stutter insertion in haplotype *X* (insertion size Δ*L*), we assume the inserted sequence arises from a local duplication of adjacent sequence. Consequently, the duplication can originate from either the left or right flank of the insertion site. Due to boundary constraints—insertions within the first Δ*L* positions can only be duplicated from the right flank, and those within the last Δ*L* positions only from the left—only one duplication direction is considered for such edge cases. The alignment likelihood is determined jointly by the stutter insertion rate and the base-wise agreement between the read and the haplotype. As before, the overall likelihood is obtained by averaging over all possible insertion positions and duplication directions:

(12) $$P(X,Y)=\left(u*\rho *{\left(1-\rho \right)}^{\frac{\text{Δ}L}{L_{RU}}-1}\text{*}\frac{1}{\left(L+1\right)}\right)\text{*}\sum \{\begin{array}{l} \sum_{k=0}^{\text{Δ}L-1}\left(\prod_{i=1}^{k+\text{Δ}L}Q\left(X_{i},Y_{i}\right)\right)\text{*}\left(\prod_{i=k+1}^{L}Q\left(X_{i},Y_{i+\text{Δ}L}\right)\right)\\ \sum_{k=\text{Δ}L}^{L-\text{Δ}L}\left(\prod_{i=1}^{k}Q\left(X_{i},Y_{i}\right)\right)\text{*}\left[\frac{\prod_{i=k+1}^{k+\text{Δ}L}Q\left(X_{i},Y_{i}\right)\text{*}\prod_{i=k+1}^{k+\text{Δ}L}Q\left(X_{i-\text{Δ}L},Y_{i}\right)}{2}\right]\text{*}\left(\prod_{i=k+1}^{L}Q\left(X_{i},Y_{i+\text{Δ}L}\right)\right)\\ \sum_{k=L-\text{Δ}L+1}^{L}\left(\prod_{i=1}^{k}Q\left(X_{i},Y_{i}\right)\right)\text{*}\left(\prod_{i=k+1}^{k+\text{Δ}L}Q\left(X_{i-\text{Δ}L},Y_{i}\right)\right)\text{*}\left(\prod_{i=k+1}^{L}Q\left(X_{i},Y_{i-\text{Δ}L}\right)\right) \end{array}$$

Where:

- Δ*L* denotes the insertion size in read *Y*.
- *L_RU_* denotes the motif length.
- *ρ* denotes the success probability of the geometric model, which belongs to the interval (0,1].
- *k* denotes the insertion position in the haplotype *X*.
- Other parameters are defined in Equation (10).

We use alignment likelihoods to estimate the probability that each read originates from a given haplotype, thereby determining the most likely haplotype of origin for each read (Extended Data Fig. 1d). The mutation type (insertion, deletion, or mismatch) was further determined by pairwise alignment of the inferred mutant haplotype to the germline haplotype using pairwise aligner^5^ (v1.81). This refinement alignment incorporated 4-bp flanking sequences on each side.

### Estimating global whole-genome amplification bias

To estimate whole-genome allelic balance for each single cell, we modeled alternative allele counts using a beta-binomial distribution. Germline heterozygous SNPs (hSNPs) were defined as sites with matched-bulk VAF from 0.4 to 0.6 and present in dbSNP. The two shape parameters of the Beta distribution, *α* and *β* were estimated as follows:

(13) $$\alpha =\frac{dp\text{*}m_{1}-m_{2}}{dp\text{*}\left(\frac{m_{2}}{m_{1}}-m_{1}-1\right)+m_{1}}$$

(14) $$\beta =\frac{\left(dp-m_{1}\right)\text{*}\left(dp-\frac{m_{2}}{m_{1}}\right)}{dp\text{*}\left(\frac{m_{2}}{m_{1}}-m_{1}-1\right)+m_{1}}$$

where:

- $m_{1}=\mathrm{mean}\left(n_{alt}\right)$.
- $m_{2}=\frac{\sum_{m=1}^{n_{\mathrm{site}}}n_{alt,m}^{2}}{n_{site}}$
- *dp* is defined as the median depth of hSNP loci.

Notably, the hSNPs collected for calculating allele imbalance parameters were all of the same median depth, such as 20x. *n_alt_* refers to the alternative read count, and *n_site_* refers to the total number of hSNP loci.

### Estimating local amplification bias using a piecewise gaussian process regression

In whole-genome amplification, uneven amplification across DNA fragments creates locally correlated allelic bias. We addressed this by segmenting reads into fragments using an HMM and then applying piecewise Gaussian process regression to quantify bias within each segment.

To model the correlation of allelic balance, we implemented a piecewise Gaussian process regression framework. First, reads within individual cells were partitioned into independent segments using a hidden Markov model (HMM). Within each segment, the haploid allelic fraction (AF_h_) values of the same haplotype h, derived from phased hSNPs (phased with Eagle2 as described previously), along with their genomic coordinates, served as inputs for the regression. The Gaussian process is formally defined as:

(15) $$B_{h}=f(x)∼GP\left(\mu =0,K\left(x,x^{\prime }\right)\right)\;K\left(x,x^{\prime }\right)=\sigma_{frag}^{2}\text{exp}\left(-\frac{{\left(x-x^{\prime }\right)}^{2}}{2l_{frag}^{2}}\right)$$

and the allele fraction is represented using a logistic transform:

(16) $$AF_{h}=\frac{1}{1+e^{-B_{h}}},\;B_{h}\in (-\infty ,+\infty )$$

Where:

- allele imbalance *B_h_* given hSNPs is modelled by Gaussian process *GP* with zero mean function.
- the radial basis function *K*(*x,x*′) with parameter *σ* and *l* accounting for fragments attribution.

To prevent discontinuities in the regression line caused by allele dropout, the haploid allelic fraction (AF_h_) was constrained to the interval (10^−7^, 1 – 10^−7^). This model was subsequently applied to predict AF values at each candidate STR loci. Genomic regions containing phased SNPs were excluded if their correlation curves showed sharp fluctuations, specifically those with a mean squared error (MSE) exceeding 0.5, to filter out intervals with potential phasing errors.

### Calculation of mosaic genotyping likelihood with bulk sequencing data

We made no specific assumption about the allele frequencies of mosaic mutations; instead, we adopted a uniform prior distribution over the interval [0, 1]. Based on this, the mosaic genotyping likelihood given bulk sequencing data can be calculated with the following formula:

(17) $$P\left(D|G_{i}=mosaic\right)=\int_{0}^{1}{\left(\theta \right)}^{r}{\left(1-\theta \right)}^{depth-r}d\theta =\left(\begin{array}{c} depth\\ r \end{array}\right)\beta (r+1,depth-r+1)$$

Where:

- *r* indicates alternative read count in the bulk sequencing data.
- *depth* indicates total read depth in the bulk seq data.
- *θ* indicates the prior distribution of variant allele frequency of mosaic mutations.

### Joint Genotyping

Based on population allele frequencies, sequencing reads and stutter error profiles, BayesMonSTR calculated genotyping posteriors of candidate mosaic STR mutations with a haplotype-based EM-algorithm.

#### Parameter initialization:

Population frequencies *f_a_* were set to be with equal probability. Haplotype phasing probability *P*(*j* – *h*_1_, *k* – *h*_2_|*G*_*i*_) was set to 0.5. *μ_msi_* was set to 0.001. Stutter error parameters *u*, *d* and *ρ* for each site were calculated with previous procedures.

#### E-step:

BayesMonSTR employed a hierarchical Bayesian network to incorporate population, bulk and single cell-level data. We utilized an EM algorithm to calculate maximum likelihood of latent parameters.

Similar to the length-based EM procedure, the E-step for estimating individual germline genotypes at sequence-level resolution was performed as follows:

(18) $$P\left(G_{i}^{t}=(j,k)|D_{c},D_{\mathrm{bulk}}\right)=P\left(G_{i}^{t}=(j,k)\right)\text{*}P\left(D_{bulk}|G_{bulk}^{t}=(j,k)\right)\text{*}\prod_{c=1}^{n_{cells,i}}\sum_{G_{c}^{t}}\left[P\left(G_{c}^{t}|G_{i}^{t}=(j,k)\right)\text{*}P\left(D_{c}|G_{c}^{t}\right)\right]=\;2f_{j}^{t}f_{k}^{t}\text{*}\prod_{m=1}^{n_{reads,bulk}}\sum_{a\in \{j,k\}}\frac{1}{2}P\left(r_{m,i},Q_{m,i}|S_{m,i}=a\right)\text{*}\prod_{c=1}^{n_{cells,i}}\sum \{\begin{array}{l} \sum_{\neg k\in A_{i}\backslash \{k\}}\mu_{i,k\to \neg k}^{t}\text{*}\left(1-\sum_{\neg j\in A_{i}\backslash \{j\}}\mu_{i,j\to \neg j}^{t}\right)\text{*}P\left(D_{c}|G_{c}^{t}=(j,\neg k)\right)\\ \left(1-\sum_{\neg k\in A_{i}\backslash \{k\}}\mu_{i,k\to \neg k}^{t}\right)*\left(1-\sum_{\neg j\in A_{i}\backslash \{j\}}\mu_{i,j\to \neg j}^{t}\right)\text{*}P\left(D_{c}|G_{c}^{t}=(j,k)\right)\\ \sum_{\neg j\in A_{i}\backslash \{j\}}\mu_{i,j\to \neg j}^{t}*\left(1-\sum_{\neg k\in A_{i}\backslash \{k\}}\mu_{i,k\to \neg k}^{t}\right)\text{*}P\left(D_{c}|G_{c}^{t}=(\neg j,k)\right) \end{array}$$

If the germline genotype is homozygous, the prior probability of *G_i_* is $f_{j}^{2}$ instead of 2*f_j_f_k_*.

Equation 2 and 19 were used for unphased and phased genotype likelihood calculation, respectively.

(19) $$P\left(D_{c}|G_{c}^{t}=(j,k)\right)=\prod_{m=1}^{n_{reads,c}}\sum \{\begin{array}{l} P\left(O_{m,c}|S_{m,c}=j,S_{m,c}=h_{1}\right)\text{*}P_{j-h_{1}}^{t}\\ P\left(O_{m,c}|S_{m,c}=j,S_{m,c}=h_{2}\right)\text{*}P_{j-h_{2}}^{t}\\ P\left(O_{m,c}|S_{m,c}=k,S_{m,c}=h_{1}\right)\text{*}P_{j-h_{2}}^{t}\\ P\left(O_{m,c}|S_{m,c}=k,S_{m,c}=h_{2}\right)\text{*}P_{j-h_{1}}^{t} \end{array}\;=\prod_{m=1}^{n_{reads,c}}\sum \{\begin{array}{l} AF_{c,j-h_{1}}\text{*}P\left(r_{m,c}|a=j\right)\text{*}P_{j-h_{1}}^{t}\\ AF_{c,j-h_{2}}\text{*}P\left(r_{m,c}|a=j\right)\text{*}P_{j-h_{2}}^{t}\\ AF_{c,k-h_{1}}\text{*}P\left(r_{m,c}|a=k\right)\text{*}P_{j-h_{2}}^{t}\\ AF_{c,k-h_{2}}\text{*}P\left(r_{m,c}|a=k\right)\text{*}P_{j-h_{1}}^{t} \end{array}$$

Where:

- *AF_c,j–h_1__* denotes the predicted allele fraction based on STR allele *j* at the locus for cell *c*.
- *P_j–h_1__* denotes the phasing probability that STR allele *j* is linked with germline hSNP *h*_1_.
- *P*(*r_m,c_*|*a* = *j*) denotes the read likelihoods given allele *j* based on the stutter error model.

#### M-step:

We calculated the phasing probabilities that quantify the phasing relationships between STR loci and hSNPs, which was initialized with 0.5. If STR allele *j* linked with germline SNP *h*_1_ and allele *k* links with germline SNP *h*_2_, the phasing probability given individual *i* read information *D_i_* was calculated with the following equation:

(20) $$P^{t+1}\left(j-h_{1},k-h_{2}|D_{i}\right)=\sum_{G_{i}^{t}}P^{t}\left(j-h_{1},k-h_{2}|D_{c},D_{bulk},G_{i}^{t}\right)\text{*}P\left(G_{i}^{t}\right)\;=\sum_{G_{i}^{t}}\frac{P\left(j-h_{1},k-h_{2}|G_{i}^{t}\right)\text{*}P\left(G_{i}^{t}\right)\text{*}P\left(D_{bulk}|j-h_{1},k-h_{2},G_{i}^{t}\right)\text{*}P\left(D_{c}|j-h_{1},k-h_{2},G_{i}^{t}\right)}{P\left(D_{c},D_{bulk}|G_{i}^{t}\right)}\;=\sum_{G_{i}^{t}}\frac{P\left(j-h_{1},k-h_{2}|G_{i}^{t}\right)\text{*}P\left(G_{i}^{t}\right)\text{*}P\left(D_{bulk}|j-h_{1},k-h_{2},G_{i}^{t}\right)\text{*}\prod_{c=1}^{n}[P(D_{c}|j-h_{1},k-h_{2},G_{c}^{t}=(j,k))\text{*}P(G_{c}^{t}=(j,}{P\left(D_{bulk}|G_{i}^{t}\right)\text{*}\prod_{c=1}^{n}\left[P\left(G_{c}^{t}=(j,k)|G_{i}^{t}\right)\text{*}P\left(D_{c}|G_{c}^{t}=(j,k)\right)\right]}$$

Where:

- $P(G_{i}^{t})$ denotes the population haplotype frequency prior.
- *p*(*D* | *j* − *h*_1_, *k* − *h*_2_) is the genotype likelihood for individual *i* given allele *j* linked with SNP *h*_1_ and allele *k* links with SNP *h*_2_, and with *P*(*j* − *h*_1_, *k* – *h*_2_|$G_{i}^{t}$) set to 0.5.
- $P(G_{c}^{t}=(j,k)|G_{i}^{t})$ denotes the mosaic mutation probability that the cell genotype is (*j, k*) given *G_i_*.

Similarly, the probability that STR allele *j* is linked with germline SNP *h*_2_ follows the same approach. Of note, we only used reads covering both the candidate locus and the nearby hSNP to calculate the phasing probability.

The mosaic allele fraction for mutant allele ¬*k* was calculated using the following function:

(21) $$\mu_{i,k\to \neg k}^{t+1}=\frac{\sum_{j\in A_{i}\backslash \{k\}}P\left(G_{i}^{t}=(j,k)\right)\sum_{c}P\left(G_{i}^{t}=(j,\neg k)|G_{i}^{t}=(j,k)\right)+P\left(G_{i}^{t}=(k,k)\right)\sum_{c}P\left(G_{i}^{t}=(k,\neg k)|G_{i}^{t}=(k,k)\right)}{\sum_{j\in A_{i}\backslash \{k\}}P\left(G_{i}^{t}=(j,k)\right)\sum_{c}1+P\left(G_{i}^{t}=(k,k)\right)\sum_{c}2}$$

Where:

- ($G_{i}^{t}=(j,k)$) denotes the individual germline probability in E-step.
- denotes the germline allele.

The sequence-based STR allele population frequency was calculated as follows:

(22) $$f_{j}^{t+1}=\frac{1}{2N_{ind}}\sum_{i=1}^{N_{ind}}\sum_{k=1}^{A_{i}}\left(P\left(G_{i}^{t}=(j,k)|G_{c,i}^{t},D_{c,i},D_{bulk,i},\theta_{stutter}^{t}\right)+P\left(G_{i}^{t}=(k,j)|G_{c,i}^{t},D_{c,i},D_{bulk,i},\theta_{stutter}^{t}\right)\right)$$

Where:

- All parameters are defined in Equation (18).

### Estimating somatic genotyping posteriors for each site across cells:

BayesMonSTR employed a dynamic programming approach to compute the posterior probability that a candidate locus contains at least one mutant cell. The posterior probability that a mosaic mutation occurs (*k* > ¬*k*) is calculated with the following formula:

(23) $$P_{i,k\to -k}=1-P\left(L_{i,k\to -k}=0|{\bar{D}}_{c}\right)$$

Where:

- *L_i_* denotes the mutant allele count for individual *i*.

The calculation follows with Bayes’ rule:

(24) $$P\left(L|{\bar{D}}_{c}\right)=\frac{P(L)\text{*}P\left({\bar{D}}_{c}|L\right)}{P\left({\bar{D}}_{c}\right)}\;=\frac{P(L)\text{*}\sum_{{\vec{G}}_{c}}P\left({\bar{D}}_{c}|{\vec{G}}_{c},L\right)P\left({\vec{G}}_{c}|L\right)}{\sum_{L^{\prime }=0}^{N}P\left(L^{\prime }\right)\sum_{{\vec{G}}_{c}^{\prime }}P\left({\bar{D}}_{c}|{\vec{G}}_{c}^{\prime },L^{\prime }\right)P\left({\vec{G}}_{c}|L^{\prime }\right)}\;=\frac{P(L)\text{*}\sum_{{\vec{G}}_{c}}P\left({\bar{D}}_{c}|{\vec{G}}_{c}\right)P\left({\vec{G}}_{c}|L\right)}{\sum_{L^{\prime }=0}^{N}P\left(L^{\prime }\right)\sum_{{\vec{G}}_{c}^{\prime }}P\left({\bar{D}}_{c}|{\vec{G}}_{c}\right)P\left({\vec{G}}_{c}|L^{\prime }\right)}\;=\frac{P(L)\text{*}\sum_{{\vec{G}}_{c}}P\left({\bar{D}}_{c}|{\vec{G}}_{c}\right)\delta_{L,S\left({\vec{G}}_{c}\right)}\frac{1}{\left(\begin{array}{l} N\\ L \end{array}\right)}}{\sum_{L^{\prime }=0}^{N}P\left(L^{\prime }\right)\sum_{{\vec{G}}_{c}^{\prime }}P\left({\bar{D}}_{c}|{\vec{G}}_{c}\right)\text{*}\delta_{L^{\prime },S\left({\vec{G}}_{c}^{\prime }\right)}}\;=\frac{\frac{1}{\left(\begin{array}{l} N\\ L \end{array}\right)}\text{*}P(L)\text{*}\sum_{{\vec{G}}_{c}}\delta_{L,S\left({\vec{G}}_{c}\right)}\prod_{c}P\left(D_{c}|G_{c}\right)}{\frac{1}{\left(\begin{array}{l} N\\ L \end{array}\right)}\text{*}\sum_{L^{\prime }=0}^{N}P\left(L^{\prime }\right)\sum_{{\vec{G}}_{c}^{\prime }}\delta_{L^{\prime },S\left({\vec{G}}_{c}^{\prime }\right)}\prod_{c}P\left(D_{c}|{G^{\prime }}_{c}\right)}$$

The Kronecker delta function, denoted as *δ_L,S_* is a mathematical function that evaluates to 1 when its indices are equal, and evaluates to 0 when its indices differ.

(25) $$P\left(L_{i,k\to \neg k}\right)=\left(\begin{array}{c} N_{c}\\ L_{i,k\to \neg k} \end{array}\right)\mu_{i,k\to \neg k}^{L_{i,k\to \neg k}}{\left(1-\mu_{i,k\to \neg k}\right)}^{N_{c}-L_{i,k\to \neg k}}$$

The recursive formula of dynamic programming is as follows:

(26) $$M_{l,j}=\{\begin{array}{ll} \sum_{{\vec{G}}_{c}}P\left({\bar{D}}_{c}|{\vec{G}}_{c}\right)\delta_{l,S\left({\vec{G}}_{c}\right)}\prod_{c=1}^{j}P\left(D_{c}|G_{c}\right) & 0\leq l\leq j\\ 0 & l>j \end{array}$$

(27) $$M_{l,j}=M_{l,j-1}\text{*}P\left(D_{j}|G_{j}=(j,k)\right)+M_{l-1,j-1}\text{*}P\left(D_{j}|G_{j}=(j,-k)\right)$$

Where:

- *M_l,j_* denotes probability for mutant allele count *l* and allele *j* in the DP matrix.

### Haplotype phasing related artifacts

Haplotype phasing is widely adopted as a methodology to increase confidence of mosaic mutation calling^6^. The probabilistic haplotype phasing approach we designed is based on two key assumptions: 1) Given a relatively low mutation rate, it is highly unlikely that somatic mutations would occur independently on both haplotypes at the same position; 2) non-cancer cells possess diploid genomes.

Assuming STR allele *j* is linked with hSNP *h*_1_, and STR allele *k* is linked with hSNP *h*_2_. Discordant rate of reads for bulk sample *d_bulk_* and single cell sequencing data *d_sc_* were defined as

(28) $$d_{bulk}=\frac{n_{k-h_{1}}}{n_{j-h_{1}}+n_{k-h_{1}}}$$

(29) $$d_{sc}=\frac{n_{k-h_{1}}}{n_{j-h_{1}}+n_{k-h_{1}}}$$

Amplification error *d_amp_* rate and discordant rate *d_k_* for mutant cells sample were defined as:

(30) $$d_{amp}=\frac{n_{j-h_{2}}}{n_{j-h_{2}}+n_{k-h_{2}}}$$

(31) $$d_{k}=\frac{n_{k-h_{1}}}{n_{k-h_{1}}+n_{k-h_{2}}}$$

Where:

- *n*_*j–h*_1__ is the number of reads that STR allele *j* is linked with hSNP *h*_1_.
- *n*_*k–h*_1__ is the number of reads that STR allele is linked with hSNP *h*_1_.
- *n*_*j–h*_2__ is the number of reads that STR allele *j* is linked with hSNP *h*_2_.
- *n*_*k–h*_2__ is the number of reads that STR allele *k* is linked with hSNP *h*_2_.

Based on empirical assessment, candidate mutations are considered high-confidence somatic events when all four of their phasing error rates (defined in Equations 28–31) are below 0.1.

### Genome-wide variant prediction using Random Forest (RF) classification model

We trained two Random Forest classifiers to predict mosaic variants using a rigorously curated training set with phasing information (Supplementry Table 2). The first model incorporated phasing-related features, while the second model was designed for genome-wide prediction, regardless of phasability. A leave-one-donor-out strategy was applied in model training and testing.

The model was specifically trained to predict refined genotypes, categorizing sites into five distinct classifications: amplification error (amp error), sequencing error (seq error), repeat, heterozygous (het) and mosaic. To ensure an unbiased performance assessment, we employed a leave-one-out cross-validation strategy for model training and testing. To identify mosaic loci, we applied a dual-mode filtering strategy based on phasing feasibility. Phase-informed mode: Variants initially predicted by the phase Random Forest model underwent a series of phasing-based hard filters to yield a high-confidence set of phasing-corrected mosaic loci. These filters included thresholds for bulk, single-cell, and mutant-cell discordance rates, alongside an amplification error rate (all < 0.1, as defined in Equations 28–31), supplemented by requirements for mutant cell VAF ≥ 0.1 and a mutant read count ≥ 2. Non-phaseable mode: For variants lacking reliable phase information, two sequential Random Forest models were applied to mitigate amplification errors. To derive a stringent set of true mosaic sites, we then applied an additional empirical filter requiring mutant cell VAF > 0.25 and mutant read count ≥ 3.

### Examination of artifact sites identified by different software tools

To investigate the superior performance of BayesMonSTR over HipSTR and ExpansionHunter in detecting mosaic STR mutations from single-cell data, we systematically analyzed the false-positive calls from the latter two tools. This analysis revealed how BayesMonSTR’s multi-step filtering pipeline eliminates such artifacts. The key filtering steps are defined as follows: 1. Prescan: Candidate loci lacking alternative alleles or residing in low-depth regions (mean depth ≤ 1 across cells) were removed. 2. Genotyping: Candidate mutations with a mosaic genotyping posterior probability below 0.9 were excluded. 3. Phasing: Candidate mutations were filtered based on the phasing metrics defined in Equations 28–31. Specifically, we excluded any mutation with a bulk discordant rate, single-cell discordant rate, amplification error rate, or mutant-cell discordant rate exceeding 0.1. 4. RF model: Candidate mutations were classified as mosaic if the highest predicted probability was for the mosaic class. The SHAP^7^ (SHapley Additive exPlanations, SHAP, v0.46.0) method was then used to identify the key features driving the RF classification for these mutations (Extended Data Fig. 4e).

### Simulation of spike-in mosaic STR mutations in single cell sequencing data

To simulate mosaic STR mutations in single cell sequencing data, STR indels and mismatches were randomly introduced at target loci using BAMSurgeon^8^ (v1.4.1). Diploid single-cell data were then sampled via beta-binomial sampling to emulate allele-specific amplification imbalance. Finally, aligned reads from 10 single-cell libraries (male donors) were down-sampled and merged to generate synthetic diploid datasets for validation.

### Breast cancer sample collection

A fresh breast cancer tissue sample and a matched normal breast tissue were obtained from a female breast cancer patient at The Second Affiliated Hospital Zhejiang University School of Medicine. The study was approved by the Institutional Review Board of Westlake University (IRB: 20230213DYM001). Informed consent was obtained from the patient in accordance with the IRB-approved protocol, and consent for data publication was also provided. At the time of collection, pathologist analysed the samples and categorized them into tumor and matched normal breast tissue blocks. Fresh tissue samples were transferred to sterile PBS buffer (Sangon Biotech, E607008) on ice and processed immediately upon arrival at the laboratory. The breast cancer and normal tissue samples were then dissociated, and viable single cells were isolated. Single-cell whole genome amplification, library preparation, and whole-genome sequencing were subsequently performed as described below.

### Isolation of single cells of breast tissue samples for scWGS

Fresh tumor and matched normal breast tissue samples were collected and processed within two hours post-surgery. For each sample, small tissue blocks of approximately 1 cm^3^ were isolated for dissociation. The tissue dissociation procedure was performed according to previously described methods^9,10^, with slight modifications. Briefly, tissue samples were manually minced in 1.5 mL tubes containing collagenase type 3 (300 U/mL) (Worthington Biochemical, #LS004182) and hyaluronidase (100 U/mL) (Worthington Biochemical, #LS002592) for 2 hours, with gentle pipetting every 60 minutes. The digested tissue was then centrifuged at 1500 rpm for 5 minutes. The pellet was digested with 5 mL of pre-warmed 0.25% trypsin-EDTA (Thermo #25200072) at 37°C for 5 minutes. Digested cells were washed once with HBSS + 2% FBS (Jackson, #017-000-121), then treated with 0.1 mg/mL DNase I (Worthington Biochemical, #LS002139) in DMEM/F12 media at 37°C for 5 minutes. After washing, the cells were filtered through a 40 μm mesh. Dissociated cells were counted and resuspended in the designated medium for subsequent staining and FACS sorting. Meanwhile, tissue surrounding the small blocks (within 0.5 cm from the edge) was similarly dissociated into a single-cell suspension. DNA was then extracted using a DNeasy Blood & Tissue Kit (Qiagen, 69504) from these suspensions for bulk DNA whole genome sequencing. For FACS sorting, single cells were stained with the following antibodies: CD45 (BioLegend, 304022), CD31 (BioLegend, 303114), CD49f (Thermo, 12-0495-82), EpCAM (BioLegend, 324204), and CD34 (STEMCELL Technologies, 60013Az.1). The antibodies were used at a dilution of 1:200, with appropriate conjugated fluorophores, and incubated for 30 minutes on ice. After staining, cells were washed and resuspended in Hanks Balanced Salt Solution (HBSS) + 2% FBS + Antibiotic-Antimycotic + 4′,6-diamidino-2-phenylindole (DAPI) (1 μg/mL) at a density of 5 million cells/mL. The stained samples were then analyzed and sorted on a FACSAria Fusion (BD Biosciences) with a 100-μm nozzle. The target single cells were sorted into individual tubes, each containing a single cell and 3 μL of cell buffer, prepared for subsequent single-cell whole genome amplification. The FACS data were further processed using FlowJo V10. Gating strategy is shown in Extended Data Fig. 4.

### Whole genome amplification and single cell DNA & RNA sequencing

The sorted single cells were subjected to whole-genome amplification using either REPLI-G (Qiagen, 150023) or ResolveOME (Bioskyrb). Single cells amplified with ResolveOME yielded both whole-genome amplification products and full-length transcriptome amplification products. In some cases, both reactions were combined to generate both whole-genome amplification products (via REPLI-G) and full-length transcriptome amplification products from the same single cell. All whole-genome and full-length transcriptome amplification products from ResolveOME underwent library preparation using the ResolveOME library preparation protocol, including a 9-10 cycle PCR library amplification. For REPLI-G whole-genome amplification products, as well as the extracted paired bulk DNA, PCR-free library preparation was performed using the Hieff NGS Ultima Pro PCR Free DNA Library Prep Kit V2 (Yeashen, 12196).

The WGS libraries were sequenced on the BGISEQ DNBSEQ-T7 platform (BGI Inc.) using a paired-end sequencing length of 150 bp (PE150). The average sequencing depth for each WGS library was 30–40x, while the bulk WGS library had an average sequencing depth of 200x. Full-length transcriptome libraries were sequenced on the NovaSeq X Plus platform (Illumina Inc.) using the PE150 strategy, generating approximately 20 million reads per library. Additionally, parallel library preparation of the same whole-genome amplification product or parallel sequencing of the same library was conducted to obtain WGS data duplication.

### Processing of RNA sequencing data

For RNA sequencing data, trimmomatic^11^ (v 0.39) was first used to remove adapters. Salmon^12^ (v 1.10.2) was used to quantify the expression of transcripts. The salmon index was constructed using the GTF and FASTA files (v GRCh37.87) from ENSEMBL.

### Comparison of algorithms detecting mosaic STR mutations from the benchmarking dataset

To adapt the two germline mutation callers for identifying mosaic STRs from single cell sequencing data, we applied a series of stringent filters to each software to generate a mosaic mutation call set.

HipSTR^3^ (v0.6.2) was run using the mode of “*de novo* stutter estimation + STR calling with *de novo* allele generation”, variant calls were then filtered following recommended empirical cutoffs (--min-call-qual 0.9 --max-call-flank-indel 0.15 --max-call-stutter 0.15 --min-call-allele-bias -2 --min-call-strand-bias -2). ExpansionHunter^13^ (v5.0.0) with default settings were used for variant callings of benchmark datasets.

Germline genotypes and mosaic genotypes were identified as the most frequent and second most frequent genotypes across all cells. Cell-specific variants were identified by those that have one mutant cell, and shared variants were identified as those with multiple mutant cells. In addition, if there are less than five valid cell calls, the candidate loci were excluded from the mutation list.

For BayesMonSTR, variants were identified using a genotyping cutoff of mosaic posterior ⩾ 0.9, predicted as mosaic by the RF model, and passing a series of hard filters. More specifically, for phasable sites, the hard filters include a series of phasing-related filters (equations 28–31, bulk discordant rate < 0.1, single cell discordant rate < 0.1, amplification error rate < 0.1 and mutant cell discordant rate < 0.1), and other filters (VAF ⩾ 0.1 and mutant read count ⩾ 2); For non-phasable sites, the hard filters include VAF > 0.25 and mutant read count ⩾ 3.

### Cross-validation of candidate mutations

To evaluate candidate loci identified by different software tools, we assessed their performance using orthogonal data: duplicate libraries from the same single cell, RNA sequencing data from the same cell, and bulk sequencing data from adjacent tissues.

In both duplicate library sequencing data and single-cell DNA&RNA data, a one-tailed binomial test (lower-tail) was applied, where n represents the total read count and k represents the mutant read count. For RNA-based validation, θ = 0.5 was used as the null hypothesis parameter. For duplicate cell validation, the test was conducted using the maximum p-value derived from tests with either the VAF in the mutant cell or θ = 0.5 as the null hypothesis parameter. Validation outcomes were classified as validated (p ≥ 0.05 and k > 0), unvalidated (p < 0.05 and k = 0), or undetermined (all other cases). Validation rates were calculated after excluding undetermined loci.

Shared variants were further evaluated by manual inspection and PhyloSOLID (v 0.0.1) STR genome validation mode (https://github.com/douymLab/PhyloSOLID). Cell genotype likelihoods along with alternative and total read depth information were used for shared variants evaluation based on phylogenetic tree.

We also assessed the mutations from DNA utilizing RNA sequencing data from the same cell (Extended Data Fig. 4a). This orthogonal validation approach enabled us to evaluate the concordance between DNA-based mosaic detection and transcript-level variation.

### Visualization of STR mutations

INSIGHT (v 0.0.1) developed by our lab were used for visualization of STR mutations (https://github.com/douymLab/INSIGHT).

### Identification of mosaic SNVs from single cells

Similar strategies were applied to detect mosaic SNVs in the same datasets. After sequence alignment and data preprocessing, raw candidate loci were identified by mpileup in SAMtools^14^ (v1.13) with the parameters ‘-B -d 8000 -q 10 -Q 10’. Then, the mutations were identified by using MosaiSC-DNA^15^ (v0.0.1, https://github.com/douymLab/mosaicSC). The predicted SNVs were further filtered with mutant cell VAF ⩾ 0.3 and mutant read count ⩾ 3 to get a more reliable set of mosaic SNVs.

### Phylogeny reconstruction using shared variants

Given the modest validation rate for shared variants, the constructed phylogenetic tree (Figure 2g) was based on manually checked cell-by-mutation matrix (Supplementry Table 5). PhyloSOLIDD^16^ (v0.0.1) was used for phylogeny reconstruction.

### Correlation of age with multiple mutation-derived features

We performed association analyses between age and multiple mutation-derived features, including mutation rate, variant length, and cell proportion with >5bp indels. Among these, the mutation rate was calculated by dividing the number of mutations by the total length of callable regions. We used scipy^17^ (v 1.14.1) to perform linear regression analysis between age and features.

### Mutation signature analysis

For mutations in STR regions, mutation matrices were generated using SigProfilerMatrixGenerator^18^ (v 1.2.31), and then, SigProfilerAssignment^19^ (v 0.1.9) was used to assign known COSMIC mutation signatures to every individual, which was accomplished using the forward stagewise algorithm and nonnegative least squares. In our samples, three 83-cat signatures of small insertions and deletions (ID1, ID2, ID12) and multiple 96-cat signatures of single base substitutions were found to be active. Additionally, SigProfilerAssignment was used to assign signature probabilities to every mutation, allowing to quantify the mutation burden associated with each signature for every individual.

Additionally, we also used a redefined InDel taxonomy^20^ to illustrate mutation profile for InDels in STR regions, and the mutation signature similarity was computed via cosine similarity was used to compute similarity between our InDels and those caused by the *MSH2* and *MSH3* knockouts^20^.

Specially, for SNVs in STR regions, a custom method was developed to classify SNVs into different categories. Initially, mutations were distinguished based on the six kinds of fundamental base changes, followed by classification according to the nucleotide context of the entire STR region in which the SNV occurred (considering both repeat unit length and total length of the repeat region). Subsequently, non-negative matrix factorization was performed using MutationalPatterns^21^ (v 3.12.0) to extract three distinct *de novo* mutation signatures. Based on the relative contribution of each signature, the mutation burden associated with each signature for every individual was quantified.

### Relationship between mosaic SNVs in DNA repair gene and mosaic STR indel burden

The list of DNA repair genes was obtained from the comprehensive compilation maintained by the laboratory of Richard D. Wood at The University of Texas MD Anderson Cancer Center (https://www.mdanderson.org/research/departments-labs-institutes/labs/wood-laboratory/resources.html). This curated gene set represents an updated synthesis based on previously published reviews.

ANNOVAR^22^ (v 2020-06-07) was used to identify exonic variants, their associated genes, and their effects on the protein sequence, including synonymous, nonsynonymous, stop-gain, stop-loss, and frameshift variants. Variants annotated as “unknown” by ANNOVAR due to incomplete ORF information were re-annotated using the Ensembl Variant Effect Predictor^23^ (VEP, https://www.ensembl.org/vep) to determine their coding consequences based on updated transcript models.

To assess the association, we fitted a generalized linear mixed model (GLMM) with a negative binomial distribution to account for overdispersion in count data. The model included somatic SNV status (presence in DNA repair gene vs. absence) as a fixed effect and a random intercept for individual to account for within-individual correlations (e.g., multiple neurons or samples per individual). The significance of fixed effects was evaluated using Wald tests. All analyses were performed in R (v 4.3.3) using the glmmTMB^24^ (v 1.1.14) package.

### Identification of potentially functional-disrupting mosaic STR mutations

ANNOVAR was employed to determine the nearest gene of mosaic STR mutations and identify potentially damaging exonic variants, including nonsynonymous, stopgain, stoploss, and frameshift variants. CADD^25^ (v 1.7) scores were incorporated to assess variant deleteriousness. ENCODE’s candidate cis-regulatory elements annotation^26^ was used to identify variants with potential regulatory functions. The chromatin state annotation from ENCODE was used to identify variants in active TSS and enhancer regions. Chromatin state annotations were derived from the ChromHMM 18-state model from the ENCODE portal^27^ for seven primary B cell samples, 17 lung tissues and 16 brain tissues.

Potentially functional-disrupting STR indels were defined as variants in upstream, UTR or exonic regions annotated by ANNOVAR, or regions defined as chromatin state of active TSS and enhancer by ENCODE, or variants have CADD score greater than 20. Gene affected by a STR indel was determined as the nearest gene annotated by ANNOVAR.

### Identification of mosaic STR mutations in functional non-coding elements

To identify STR mutations may have a significant impact on gene expression, we utilized the human G-quadruplexes prediction panel from EndoQuad^28^ for G-quadruplex forming regions, the Level 2 R-loop annotation panel from R-loop Base^29^ for R-loop forming regions, ENCODE’s candidate cis-regulatory elements annotation^26^ for CTCF-binding sites, and the CpG islands annotation from the UCSC Genome Browser^30^ for CpG islands.

### Identification of mosaic STR mutations near genes of high expression

Gene expression data of whole blood, lung and brain cortex tissues was downloaded from GTEx^31^. Within each tissue, we calculated quartiles of expression of all genes near STR loci and categorized each gene into four categories (Q1 to Q4) based on average expression level across samples, with Q1 representing expression level is in minimum to first quartile.

### Gene ontology (GO) and disease enrichment

KOBAS^32^ (v 3.0) was used to conduct Gene Ontology (GO) and Kyoto Encyclopedia of Genes and Genomes (KEGG) disease enrichment of genes near STR indels. To be specific, Fisher’s exact test was used to calculate P-values, while Benjamini and Hochberg (1995) method was used to control the FDR.

### Process of snATAC-seq data

Raw FASTQ files of snATAC-seq were aligned to the hg38 reference genome, using 10x cellranger-atac count toolkit (v 2.1.0). Only cells identified through cellranger’s cell calling were included in subsequent analyses. Duplicate reads were first filtered to ensure data integrity. Reads sharing identical cell barcodes and genomic coordinates (start and end positions) were grouped. Within each group, only reads with identical sequences were considered duplicates; among these, the read with the highest mapping quality and average base quality was retained.

### Detection of mosaic STR mutations from snATAC-seq

Building upon the framework of BayesMonSTR, we adapted the approach to better handle the sparsity of single-cell ATAC-seq data by performing genotyping at the pseudo-bulk level.

The stutter error model was trained using 26 snATAC-seq datasets derived from brain tissue and whole blood samples, obtained from the publicly available studies SRP309633 and SRP192525. After estimating the stutter error model and extracting alleles through STR segmentation as mentioned before, we directly estimated individual-level mosaic posterior probabilities at the pseudo-bulk level.

We than perform multiple strict filters to retain highly confident mutations including

1. posterior probability exceeding 0.5.
2. count of used reads in genotyping exceeding 10.
3. homozygous in the bulk genotype with exactly one alternative read.
4. no other reads at the locus contained off-target stutter artifacts or mismatches.
5. recurred in less than two individuals.

### Per-cell mutation rate estimation

In snATAC-seq data, the mutation rate per cell can be estimated for each individual cell as the ratio of the number of detected mutations to the size of its callable region. Callable regions were defined as regions covered by at least one read spanning STR with high mapping quality. And the mutation rate of an individual (*μ*) can be estimated by the mean of cells’ mutation rate:

(32) $$\mu =\frac{\sum_{i=1}^{N_{c}}\left(\frac{M_{i}}{L_{i}}\right)}{N_{c}}$$

Where:

- *M_i_*: the number of somatic mutations detected in cell *i*;
- *L_i_*: the total length (bp) of the callable genomic regions (i.e., sequenced with sufficient coverage and quality) in cell *i*;
- *N_c_*: the total number of cells analyzed.

The mutation rate of the regulatory region was calculated using the same strategy, with the exception that regulatory callable regions were used instead of whole callable regions.

### Dual-Luciferase promoter reporter assay

The promoter sequences of *NRN1* and *CNTNAP2* were PCR-amplified from HEK293T cDNA templates and cloned into our dual-luciferase reporter plasmid vector. All constructs were verified by sequencing. 0.2M HEK293T cells were seeded into 24-plate well with DMEM for 12 hours, cells were transiently transfected with plasmids using polyethyleneimine transfection reagent (1mg/mL; Polyscience, #23966-1). After 48 hours, promoter activity was analyzed using the Dual-Luciferase Reporter Gene Assay Kit (Yeasen Biotechnology, 11402ES60) following manufacturer’s protocol on a Varioskan LUX Microplate reader (Thermo). Relative promoter activity was calculated as the ratio of firefly/Renilla luminescence.

## Supplementary Material

1

## Figures and Tables

**Table 1:** Likely pathogenic exonic STR mutations identified in this study

Pos	Mutation	Cell type	Individual	Gene	Mutation type	CADD score	Polyphen2 score	Associated Disease
chr3:195254670	CT>C	B cell	A11365	*PPP1R2*	frameshift	6.348		Alzheimer’s disease
chr5:45695972	TCGOT	B cell	A19553	*HCN1*	inframe	17.83		Epilepsy
chr6:109906329	GCTT>G	B cell	BF2	*AK9*	inframe	15.01		Developmental delay
chrl2:6777069	T>TTGC	B cell	BF2	*ZNF384*	inframe	6.415		Acute myeloid leukemia
chrl :87045896	TCCTACA>T	B cell	BM2	*CLCA4*	inframe	1.747		Cystic fibrosis
chr3:32022616	C>T	B cell	A19552	*OSBPL10*	nonsynonymous	25.1	0.776	Liver disease
chrl2:54332796	G>A	B cell	A19553	*HOXC13*	nonsynonymous	16.01	0.949	Ectodermal dysplasia
chrl 9:48342606	CA>C	Neuron	UMB5559	*CRX*	frameshift	24.4		Retinitis pigmentosa
chr3:19554535	A>AG	Neuron	UMB5532	*KCNH8*	frameshift	27.5		Epilepsy
chrl9:30503438	G>GGT	Neuron	UMB5823	*URI1*	frameshift	30		Intellectual disability
chr4:47945399	TTTTTTC>T	Neuron	UMB5871	*CNGA1*	inframe	18.74		Retinitis pigmentosa
chr3:65425560	T>TCTG	Neuron	UMB5823	*MAGI1*	inframe	14.23		Autism spectrum disorder
chr2:168104768	T>A	Neuron	UMB5511	*XIRP2*	nonsynonymous	22.9	0.986	Muscular dystrophy
chrl 8:21594903	C>G	Neuron	UMB5657	*TTC39C*	nonsynonymous	21.2	0.821	Developmental delay
chrl4:65392796	C>A	Neuron	UMB1278	*CHURC1*	nonsynonymous	32		Autism spectrum disorder
chrl9:35940786	T>A	Neuron	UMB1465	*FFAR2*	nonsynonymous	27.9	1	Type 2 diabetes
chr6:127837528	GCT>G	Lung epithelium	PD34207	*SOGA3*	frameshift	24.3		Autism spectrum disorder
chr5:14487617	(GGC)_8_A>G	Lung epithelium	PD34207	*TRIO*	inframe	18.91		Autism spectrum disorder
chrl 2:51693392	T>G	Lung epithelium	PD34209	*BIN2*	nonsynonymous	16.2085	0.977	Cardiomyopathy

## Data Availability

WGS data and single-cell RNA-seq data from breast cancer samples generated in this study will be publicly available in the Sequence Read Archive (SRA). WGS data for neurons, B cells, and lung epithelium are accessible through dbGaP under accession numbers phs001485.v3.p1 and phs001808.v1.p1, and in the European Genome-phenome Archive (EGA) under accession EGAD00001005193, respectively. sn-ATAC-seq data from brain tissue are available in the EGA (accession EGAS00001001934) and Synapse (accession syn52293417).

## Code availability

BayesMonSTR is implemented in Python and R and is licensed under the MIT License. The source code, documentation and examples are available on GitHub at https://github.com/douymLab/BayesMonSTR.

## References

[1] Thorpe J., Osei-Owusu I. A., Avigdor B. E., Tupler R. & Pevsner J. Mosaicism in Human Health and Disease. Annu. Rev. Genet. 54, 1–24 (2020). 32916079 10.1146/annurev-genet-041720-093403 PMC8483770

[2] Luquette L. J. Single-cell genome sequencing of human neurons identifies somatic point mutation and indel enrichment in regulatory elements. Nat Genet 54, 1564–1571 (2022). 36163278 10.1038/s41588-022-01180-2 PMC9833626

[3] Dong X. Accurate identification of single nucleotide variants in whole genome amplified single cells. Nat Methods 14, 491–493 (2017). 28319112 10.1038/nmeth.4227 PMC5408311

[4] Zhao Y. High-resolution detection of copy number alterations in single cells with HiScanner. bioRxiv 2024.04.26.587806 (2024) doi:10.1101/2024.04.26.587806. PMC12214996 40595464

[5] Loh P.-R. Insights into clonal haematopoiesis from 8,342 mosaic chromosomal alterations. Nature 559, 350–355 (2018). 29995854 10.1038/s41586-018-0321-x PMC6054542

[6] Dileep V. Neuronal DNA double-strand breaks lead to genome structural variations and 3D genome disruption in neurodegeneration. Cell 186, 4404–4421.e20 (2023). 37774679 10.1016/j.cell.2023.08.038 PMC10697236

[7] Ellegren H. Heterogeneous mutation processes in human microsatellite DNA sequences. Nat. Genet. 24, 400–402 (2000). 10742106 10.1038/74249

[8] Rajan-Babu I.-S., Dolzhenko E., Eberle M. A. & Friedman J. M. Sequence composition changes in short tandem repeats: heterogeneity, detection, mechanisms and clinical implications. Nat. Rev. Genet. 1–24 (2024) doi:10.1038/s41576-024-00696-z. 38467784

[9] Gymrek M. Abundant contribution of short tandem repeats to gene expression variation in humans. Nat Genet 48, 22–29 (2016). 26642241 10.1038/ng.3461 PMC4909355

[10] Biezuner T. Comparison of seven single cell whole genome amplification commercial kits using targeted sequencing. Sci. Rep. 11, 17171 (2021). 34433869 10.1038/s41598-021-96045-9 PMC8387353

[11] Willems T. Genome-wide profiling of heritable and de novo STR variations. Nat Methods 14, 590–592 (2017). 28436466 10.1038/nmeth.4267 PMC5482724

[12] Dolzhenko E. ExpansionHunter: A sequence-graph based tool to analyze variation in short tandem repeat regions. Bioinformatics 35, 4754–4756 (2019). 31134279 10.1093/bioinformatics/btz431 PMC6853681

[13] Sehgal A., Ziaei-Jam H., Shen A. & Gymrek M. Genome-wide detection of somatic mosaicism at short tandem repeats. Bioinformatics btae485 (2024) doi:10.1093/bioinformatics/btae485. PMC11319640 39078205

[14] Cui Y. A genome-wide spectrum of tandem repeat expansions in 338,963 humans. Cell 187, 6411–6412 (2024). 39368475 10.1016/j.cell.2024.09.045 PMC11556180

[15] Gawad C., Koh W. & Quake S. R. Single-cell genome sequencing: current state of the science. Nat Rev Genet 17, 175–188 (2016). 26806412 10.1038/nrg.2015.16

[16] Niu B. MSIsensor: microsatellite instability detection using paired tumor-normal sequence data. Bioinformatics 30, 1015–1016 (2014). 24371154 10.1093/bioinformatics/btt755 PMC3967115

[17] Cortes-Ciriano I., Lee S., Park W.-Y., Kim T.-M. & Park P. J. A molecular portrait of microsatellite instability across multiple cancers. Nat Commun 8, 15180 (2017). 28585546 10.1038/ncomms15180 PMC5467167

[18] Park S. Clonal dynamics in early human embryogenesis inferred from somatic mutation. Nature 597, 393–397 (2021). 34433967 10.1038/s41586-021-03786-8

[19] Lodato M. A. & Walsh C. A. Genome aging: somatic mutation in the brain links age-related decline with disease and nominates pathogenic mechanisms. Hum Mol Genet 28, R197–R206 (2019). 31578549 10.1093/hmg/ddz191 PMC6872434

[20] Xia Y. INSIGHT. (2025).

[21] Huang L., Ma F., Chapman A., Lu S. & Xie X. S. Single-Cell Whole-Genome Amplification and Sequencing: Methodology and Applications. Annu Rev Genom Hum G 16, 1–24 (2015). 10.1146/annurev-genom-090413-025352 26077818

[22] Gonzalez-Pena V. Accurate genomic variant detection in single cells with primary template-directed amplification. Proc National Acad Sci 118, e2024176118 (2021). 10.1073/pnas.2024176118 PMC8214697 34099548

[23] Yang Q. PhyloSOLID: robust phylogeny reconstruction from single-cell data despite pervasive errors and extreme sparsity.

[24] Yoshida K. Tobacco smoking and somatic mutations in human bronchial epithelium. Nature 578, 266–272 (2020). 31996850 10.1038/s41586-020-1961-1 PMC7021511

[25] Zhang L. Single-cell whole-genome sequencing reveals the functional landscape of somatic mutations in B lymphocytes across the human lifespan. P Natl Acad Sci Usa 116, 9014–9019 (2019). 10.1073/pnas.1902510116 PMC6500118 30992375

[26] Michael A. L. Aging and neurodegeneration are associated with increased mutations in single human neurons. Science 359, 555–559 (2018). 29217584 10.1126/science.aao4426 PMC5831169

[27] Zhang L. Single-cell whole-genome sequencing reveals the functional landscape of somatic mutations in B lymphocytes across the human lifespan. Proc. Natl. Acad. Sci. 116, 9014–9019 (2019). 30992375 10.1073/pnas.1902510116 PMC6500118

[28] Yoshida K. Tobacco smoking and somatic mutations in human bronchial epithelium. Nature 578, 266–272 (2020). 31996850 10.1038/s41586-020-1961-1 PMC7021511

[29] Yang X. Cancer Risks Associated With Germline PALB2 Pathogenic Variants: An International Study of 524 Families. J. Clin. Oncol. 38, 674–685 (2020). 31841383 10.1200/JCO.19.01907 PMC7049229

[30] Koh G. C. C. A redefined InDel taxonomy provides insights into mutational signatures. Nat. Genet. 1–10 (2025) doi:10.1038/s41588-025-02152-y. PMC12081297 40210680

[31] Gupta S., Gellert M. & Yang W. Mechanism of mismatch recognition revealed by human MutSβ bound to unpaired DNA loops. Nat. Struct. Mol. Biol. 19, 72–78 (2012). 10.1038/nsmb.2175 PMC3252464 22179786

[32] Sfeir A. & Symington L. S. Microhomology-Mediated End Joining: A Back-up Survival Mechanism or Dedicated Pathway? Trends Biochem. Sci. 40, 701–714 (2015). 26439531 10.1016/j.tibs.2015.08.006 PMC4638128

[33] Scully R., Panday A., Elango R. & Willis N. A. DNA double-strand break repair-pathway choice in somatic mammalian cells. Nat. Rev. Mol. Cell Biol. 20, 698–714 (2019). 31263220 10.1038/s41580-019-0152-0 PMC7315405

[34] Petruseva I. O., Evdokimov A. N. & Lavrik O. I. Molecular Mechanism of Global Genome Nucleotide Excision Repair. Acta Naturae 6, 23–34 (2014). 24772324 PMC3999463

[35] Miller M. B. Somatic genomic changes in single Alzheimer’s disease neurons. Nature 1–9 (2022) doi:10.1038/s41586-022-04640-1. PMC9357465 35444284

[36] Fotsing S. F. The impact of short tandem repeat variation on gene expression. Nat. Genet. 51, 1652–1659 (2019). 31676866 10.1038/s41588-019-0521-9 PMC6917484

[37] Xiong X. Epigenomic dissection of Alzheimer’s disease pinpoints causal variants and reveals epigenome erosion. Cell 186, 4422–4437.e21 (2023). 37774680 10.1016/j.cell.2023.08.040 PMC10782612

[38] Morabito S. Single-nucleus chromatin accessibility and transcriptomic characterization of Alzheimer’s disease. Nat. Genet. 53, 1143–1155 (2021). 34239132 10.1038/s41588-021-00894-z PMC8766217

[39] Hampel H. The Amyloid-β Pathway in Alzheimer’s Disease. Mol. Psychiatry 26, 5481–5503 (2021). 34456336 10.1038/s41380-021-01249-0 PMC8758495

[40] Xue Y.-Y. CD2AP deficiency aggravates Alzheimer’s disease phenotypes and pathology through p38 MAPK activation. Transl. Neurodegener. 13, 64 (2024). 39696695 10.1186/s40035-024-00454-5 PMC11657702

[41] Ando K. PICALM and Alzheimer’s Disease: An Update and Perspectives. Cells 11, 3994 (2022). 36552756 10.3390/cells11243994 PMC9776874

[42] Du Y. MKP-1 reduces Aβ generation and alleviates cognitive impairments in Alzheimer’s disease models. Signal Transduct. Target. Ther. 4, 58 (2019). 31840000 10.1038/s41392-019-0091-4 PMC6895219

[43] Nguyen C. Transcriptional and epigenetic targets of MEF2C in human microglia contribute to cellular functions related to autism risk and age-related disease. Nat. Immunol. 1–15 (2025) doi:10.1038/s41590-025-02299-0. 41125877 PMC12571900

[44] Ellegren H. Microsatellites: simple sequences with complex evolution. Nat. Rev. Genet. 5, 435–445 (2004). 15153996 10.1038/nrg1348

[45] Wang W., Li W., Wang C., Fan W. & Dou Y. Accurate detection of mosaic mutations at short tandem repeats from bulk sequencing data. (2026).

