# Supplementary material for: Unravelling genome-wide mosaic microsatellite mutations at single-cell resolution
Source: bioRxiv. 2026 Apr 5:2026.02.04.703915. Originally published 2026 Feb 6. Preprint. [Version 2] doi: 10.64898/2026.02.04.703915 (PMC12889702; doi:10.64898/2026.02.04.703915)
Supplement: Supplement 1 [file NIHPP2026.02.04.703915v2-supplement-1.pdf]

bioRxiv preprint doi: https://doi.org/10.64898/2026.02.04.703915; this version posted April 5, 2026. The copyright holder has placed this preprint (which was not certified by peer review) in the Public Domain. It is no longer restricted by copyright. Anyone can legally share, reuse, remix, or adapt this material for any purpose without crediting the original authors.

Supplementary Figures

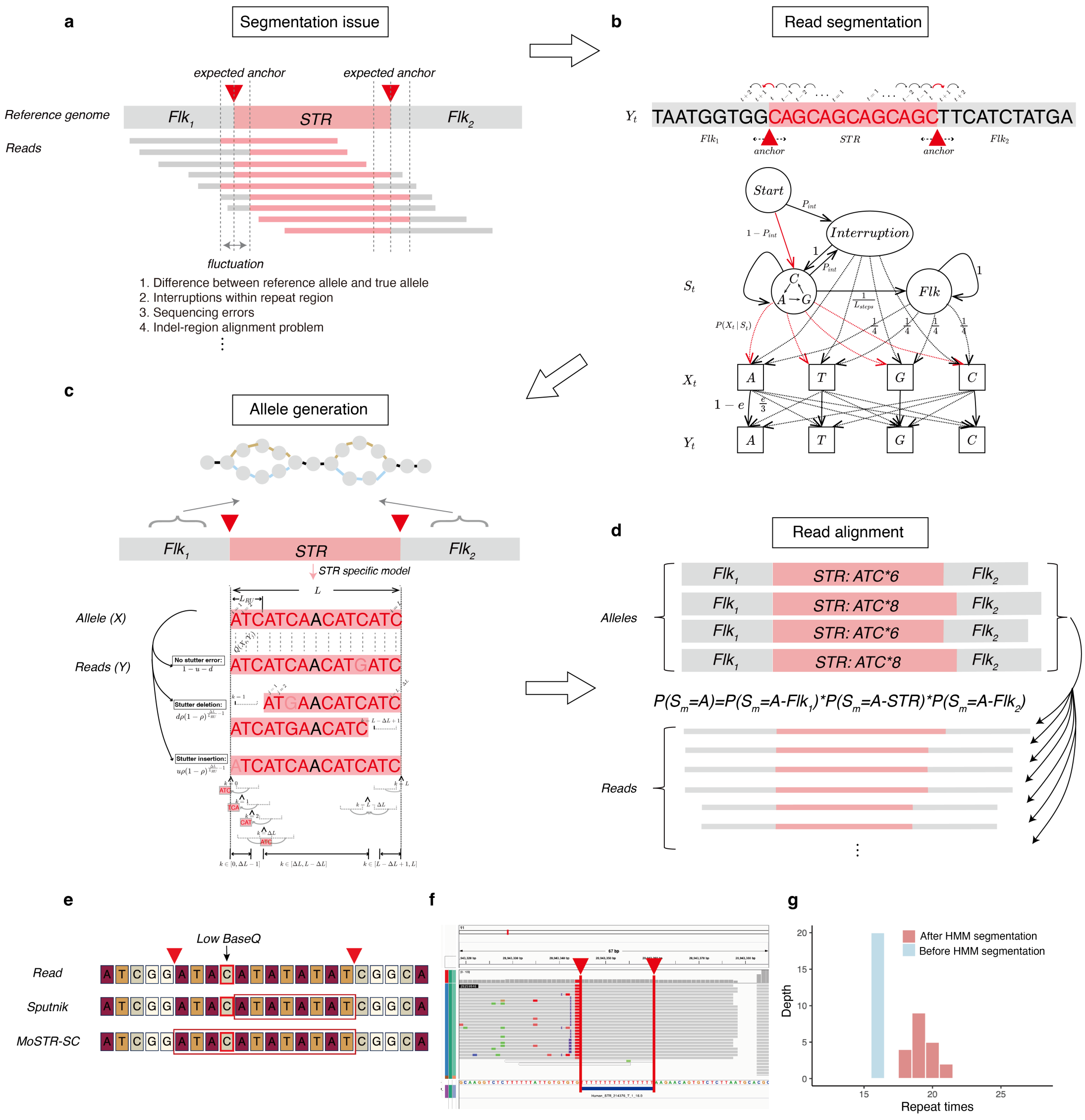

Supplementary Fig. 1 Read segmentation, allele generation and local read alignment based on sequencing reads and information from human reference genome. (a) The repeat-flank region boundary (“anchor” in the Figure) separating the STR-region and flanking regions within sequencing reads often fluctuates due to alignment issues, sequencing errors and other problems. **(b)** A fine-tuned HMM was used for reads segmentation. The three hidden states are “STR”, “flanking”, and “interruption”. Each state exhibits a preference for specific base-pair sequences and emits them with a higher probability. The segmentation boundaries between the STR and two flanking regions were identified by enumerating and selecting the optimal hidden state path. **(c)** STR-specialized local read alignment. Assuming no stutter errors occur, the read alignment likelihood was calculated by multiplying the base agreement probabilities between the read sequences and the hidden allele sequences. In the presence of a stutter error, we assume that DNA slippage occurs only once per read and that the stutter-induced INDEL can occur at any position with equal probability. **(d)** We calculate the probability that each read originates from a specific allele using an STR-specialized local read alignment strategy. **(e-g)** The read

bioRxiv preprint doi: https://doi.org/10.64898/2026.02.04.703915; this version posted April 5, 2026. The copyright holder has placed this preprint (which was not certified by peer review) in the Public Domain. It is no longer restricted by copyright. Anyone can legally share, reuse, remix, or adapt this material for any purpose without crediting the original authors.

segmentation strategy of BayesMonSTR is robust to sequencing errors and can accurately restore the correct STR length distribution despite indel fluctuations. **(e)** An example demonstrates how the scoring-based Sputnik algorithm, which ignores base quality, leads to segmentation errors. In contrast, BayesMonSTR's HMM-based segmentation strategy incorporates base quality, making it more resilient to sequencing errors. **(f-g)** Compared to BWA alignment, which often struggles with accurately defining STR-flank boundaries, the HMM-based approach of BayesMonSTR effectively tolerates indel fluctuations and restores the correct STR length distribution.

bioRxiv preprint doi: https://doi.org/10.64898/2026.02.04.703915; this version posted April 5, 2026. The copyright holder has placed this preprint (which was not certified by peer review) in the Public Domain. It is no longer restricted by copyright. Anyone can legally share, reuse, remix, or adapt this material for any purpose without crediting the original authors.

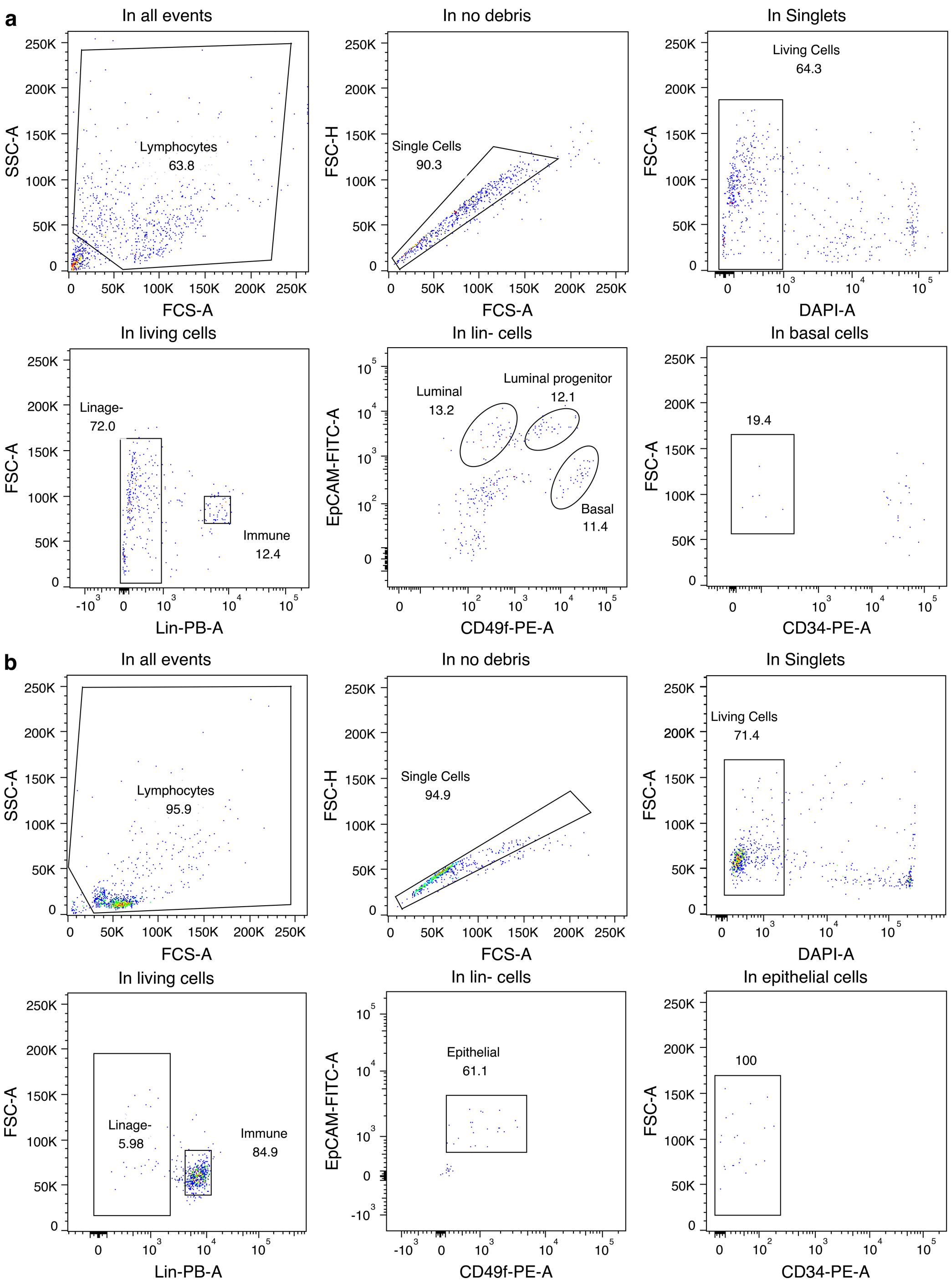

Supplementary Fig. 2 FACS gating strategy for isolating breast-tissue epithelial cells and immune cells. (a) Cell sorting results from paired normal tissue samples. Immune cells were defined as Lin+ cell populations, luminal cells were identified as Lin-EpCAM+CD49f-, luminal progenitor cells were identified as Lin-EpCAM+CD49f+, and basal cells were identified as Lin-EpCAM−CD49f+CD34− cells. **(b)** Cell sorting results from tumor samples. Immune cells were defined as Lin+ cell populations, while epithelial cells were identified as Lin-EpCAM+CD49f+CD34-. The sorting strategy was used to isolate these specific cell subsets for further analysis.

bioRxiv preprint doi: https://doi.org/10.64898/2026.02.04.703915; this version posted April 5, 2026. The copyright holder has placed this preprint (which was not certified by peer review) in the Public Domain. It is no longer restricted by copyright. Anyone can legally share, reuse, remix, or adapt this material for any purpose without crediting the original authors.

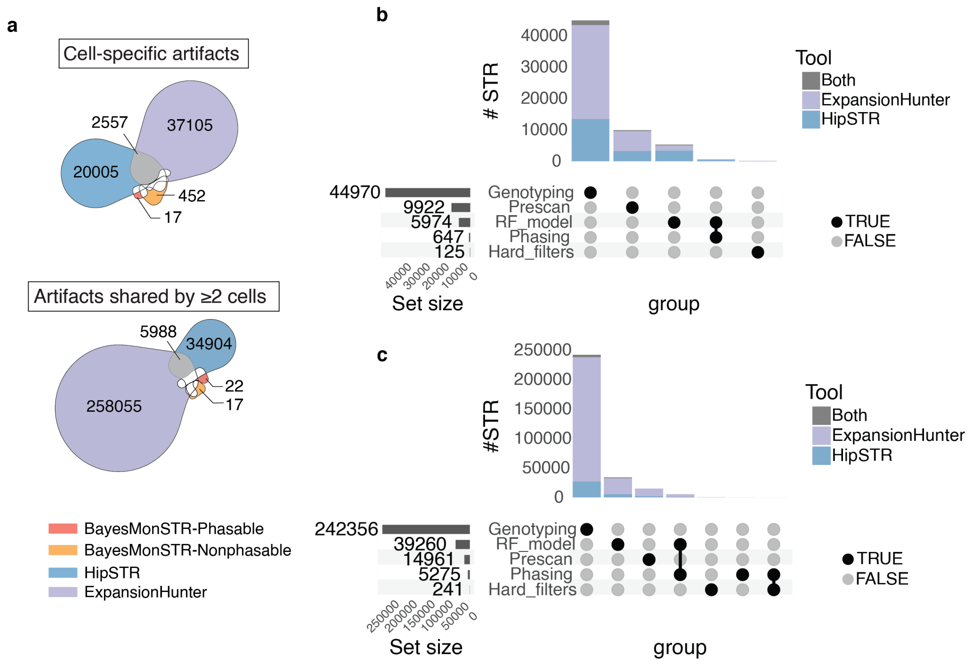

Supplementary Fig. 3 The comprehensive pipeline of BayesMonSTR pipeline effectively excludes artifacts compared with HipSTR and ExpansionHunter. (**a**) Artifact mutations identified by each method for cell-specific (top) and shared (bottom) artifacts from the benchmark dataset we generated. (**b-c**) Artifact mutations from HipSTR and ExpansionHunter were not called by BayesMonSTR through the multiple steps (Genotyping, RF model, Prescan, Phasing, Hard_filters) for cell-specific (**b**) and shared (**c**) artifacts.

bioRxiv preprint doi: https://doi.org/10.64898/2026.02.04.703915; this version posted April 5, 2026. The copyright holder has placed this preprint (which was not certified by peer review) in the Public Domain. It is no longer restricted by copyright. Anyone can legally share, reuse, remix, or adapt this material for any purpose without crediting the original authors.

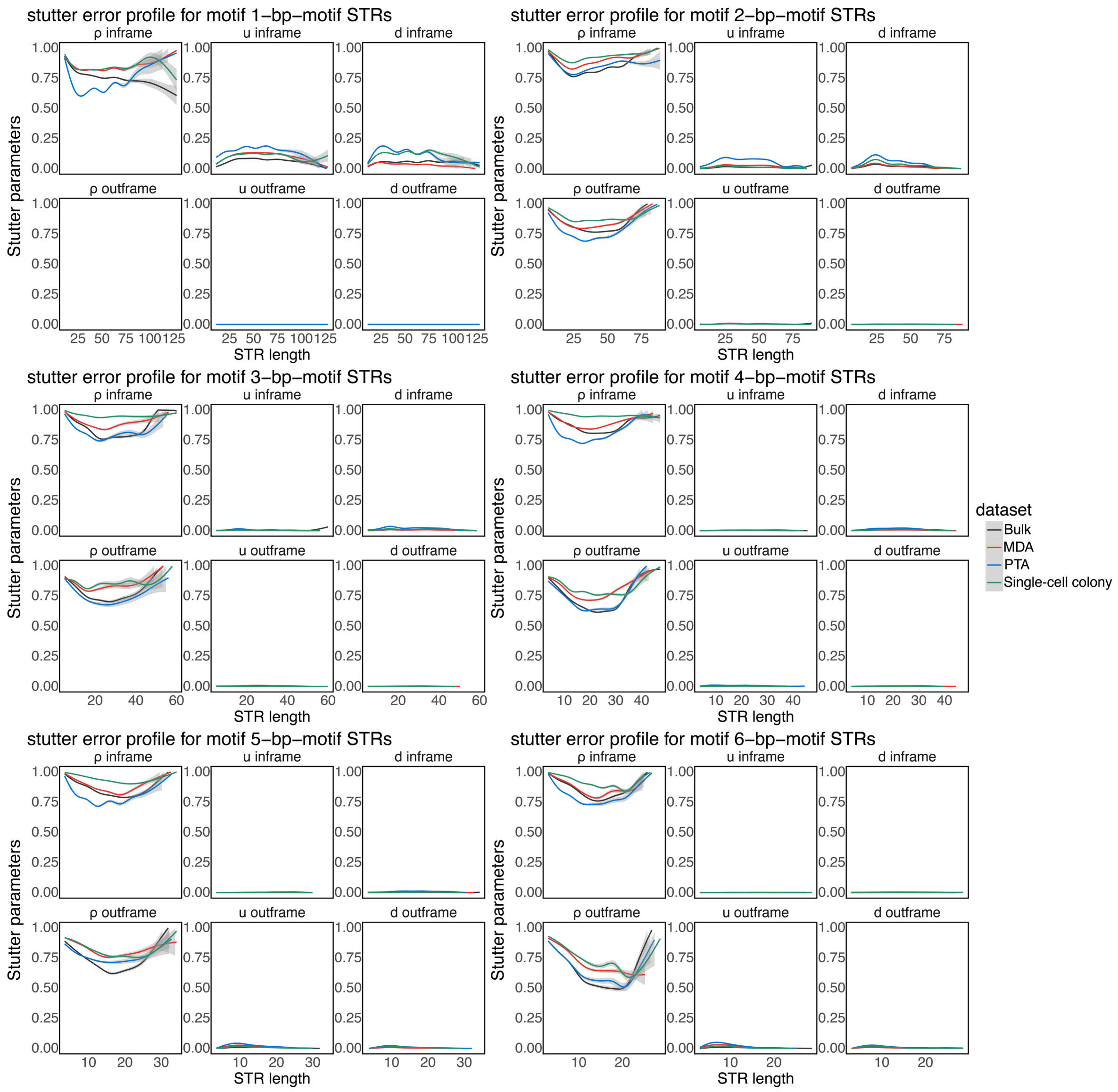

Supplementary Fig. 4 Stutter error estimation across bulk DNA sequencing data, MDA cells, PTA cells and single cell colonies. Stutter-error parameters were derived from a length-based stutter error geometric distribution model, calculated with an EM-algorithm. “Bulk” refers to bulk DNA-seq data, “MDA” refers to single-cell sequencing data amplified with multiple displacement amplification approach, “PTA” refers to single-cell sequencing data amplified with primary template-directed amplification approach, and “SCC” refers to single-cell colony data. STR loci were described by the parameters ρ (rho), u (up), and d (down), which represent the three parameters of the geometric distribution, in-frame and out-frame parameters were estimated separately (Methods). Smoothed trend lines were calculated by the generalized additive model (GAM) with cubic splines, and gray area represents 95% confidence interval.

bioRxiv preprint doi: https://doi.org/10.64898/2026.02.04.703915; this version posted April 5, 2026. The copyright holder has placed this preprint (which was not certified by peer review) in the Public Domain. It is no longer restricted by copyright. Anyone can legally share, reuse, remix, or adapt this material for any purpose without crediting the original authors.

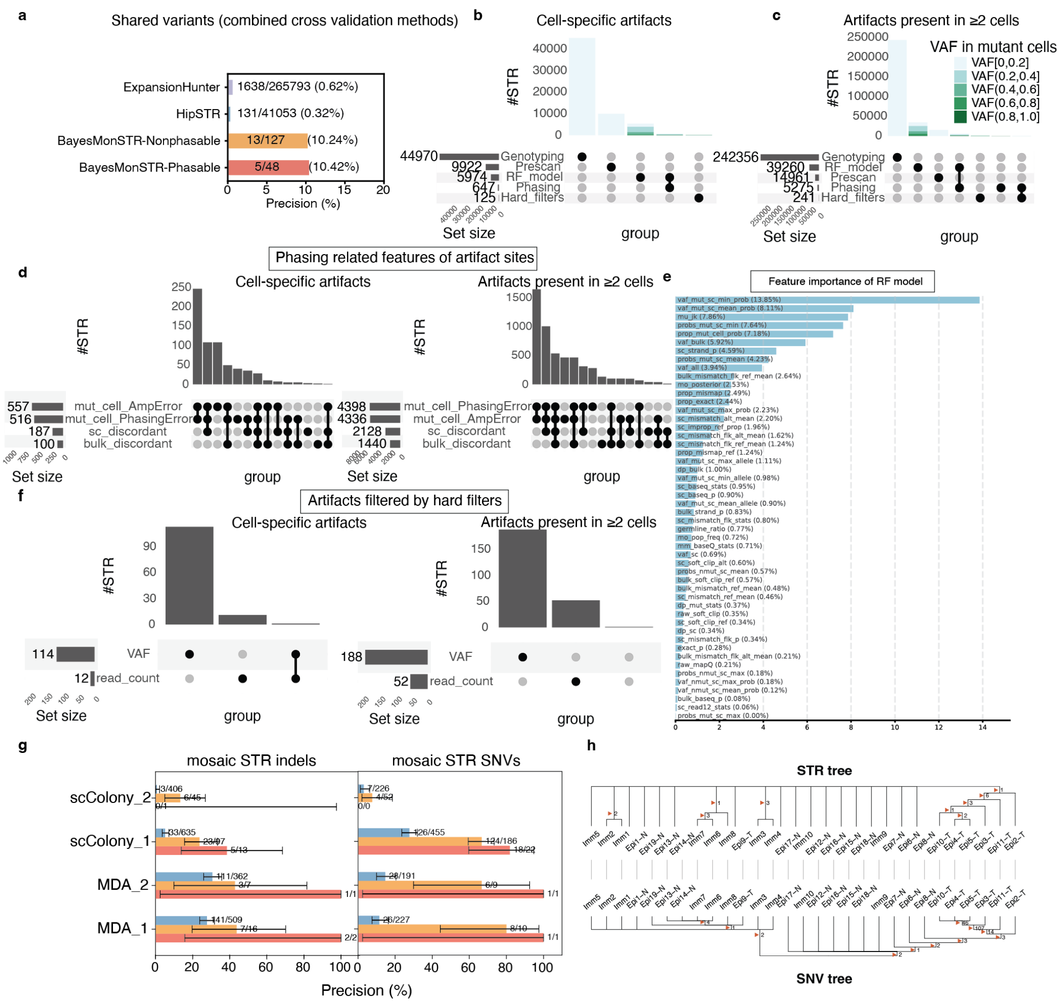

Supplementary Fig. 5 BayesMonSTR outperforms HipSTR and Expansionhunter in detecting mosaic STR mutations from single cells. (a) Validation rate of mutations presents in ≥2 cells across different software tools. The sites were extensively evaluated using both orthogonal data and checked in the phylogeny tree reconstructed with PhyloSOLID (Methods). **(b-c)** Distribution of mutant-cell VAFs for false-positive sites detected by HipSTR and ExpansionHunter. Solid points indicate false positives that were filtered by BayesMonSTR using a specific filtration method. It is evident that BayesMonSTR excluded a majority of these false positives, which had relatively low mutant allele frequencies, during the pre-scan and genotyping stages. Mutations with relatively high mutant-cell VAFs were further excluded by BayesMonSTR using the RF classification model, probabilistic haplotype phasing, and a series of hard filters. **(d)** Expanded analysis of false-positive sites identified by HipSTR and ExpansionHunter excluded by phasing-related features of BayesMonSTR. Features driving the exclusion of false positives by include discordant read pair proportion, amplification error-related features. **(e)** Key features of the random forest model of BayesMonSTR. False positives were effectively filtered by BayesMonSTR by using a combination of informative predictors. Explanations of these predictors are provided in Supplementary Table 1. **(f)** Artifact sites

bioRxiv preprint doi: https://doi.org/10.64898/2026.02.04.703915; this version posted April 5, 2026. The copyright holder has placed this preprint (which was not certified by peer review) in the Public Domain. It is no longer restricted by copyright. Anyone can legally share, reuse, remix, or adapt this material for any purpose without crediting the original authors.

excluded by BayesMonSTR using hard filters. **(g)** Performance benchmarking of BayesMonSTR versus HipSTR using additional public datasets. Error bars represent 95% confidence intervals of the validation rates calculated using binomial sampling. MDA_1 (UMB1465_21-ctx-1cP2B11_1946-WGS-PFC_19), MDA_2 (UMB1465_21-ctx-1cP2F06_1946-WGS-PFC_20), scColony_1 (10_PRL3-1_001B8_sc16), and scColony_2 (10_PRL3-2_002E11_sc17). (**h**) A phylogeny tree reconstructed using shared mutations from our benchmark shows that the mosaic STR tree roughly resembles the mosaic SNV tree. Shared STR variants were identified using only the 16 MDA cells, due to the substantially higher PCR stutter error rate inherent to PTA-amplified cells.

bioRxiv preprint doi: https://doi.org/10.64898/2026.02.04.703915; this version posted April 5, 2026. The copyright holder has placed this preprint (which was not certified by peer review) in the Public Domain. It is no longer restricted by copyright. Anyone can legally share, reuse, remix, or adapt this material for any purpose without crediting the original authors.

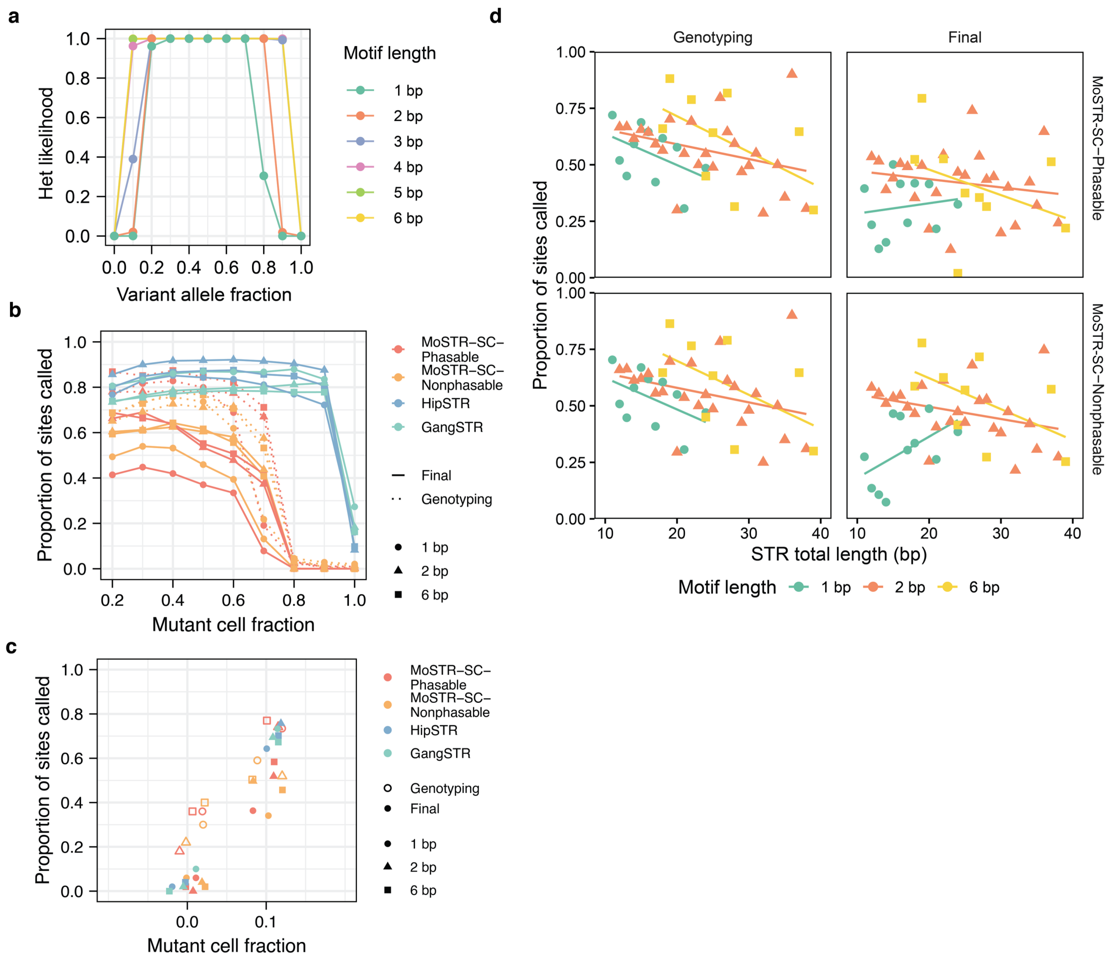

Supplementary Fig. 6 Performance evaluation of BayesMonSTR using simulated data with spike-in mutations. (a) Genotyping likelihoods of germline heterozygous at different variant allele fractions. Reads are assumed with Phred quality score 30 and median stutter error parameters were used for likelihoods calculation, heterozygous likelihoods were normalized along with reference and alternative homozygous likelihoods. **(b-c)** Sensitivity comparison across the two software tools. HipSTR was chosen for the benchmark here, given its superior performance to ExpansionHunter on the real-world data. One-motif mosaic STR insertions within 16-24 bp STR regions were generated using BAMSurgeon. The total number of cells was 10. Dashed lines indicate the raw mutation callings after genotyping of BayesMonSTR, while solid lines indicate final call sets. B illustrates the sensitivity detecting shared mutations and C illustrate cell-specific mutations. **(d)** Sensitivity variation across different STR total lengths. The total cell number was 10 and mutations spiked in were 1-motif insertions.

bioRxiv preprint doi: https://doi.org/10.64898/2026.02.04.703915; this version posted April 5, 2026. The copyright holder has placed this preprint (which was not certified by peer review) in the Public Domain. It is no longer restricted by copyright. Anyone can legally share, reuse, remix, or adapt this material for any purpose without crediting the original authors.

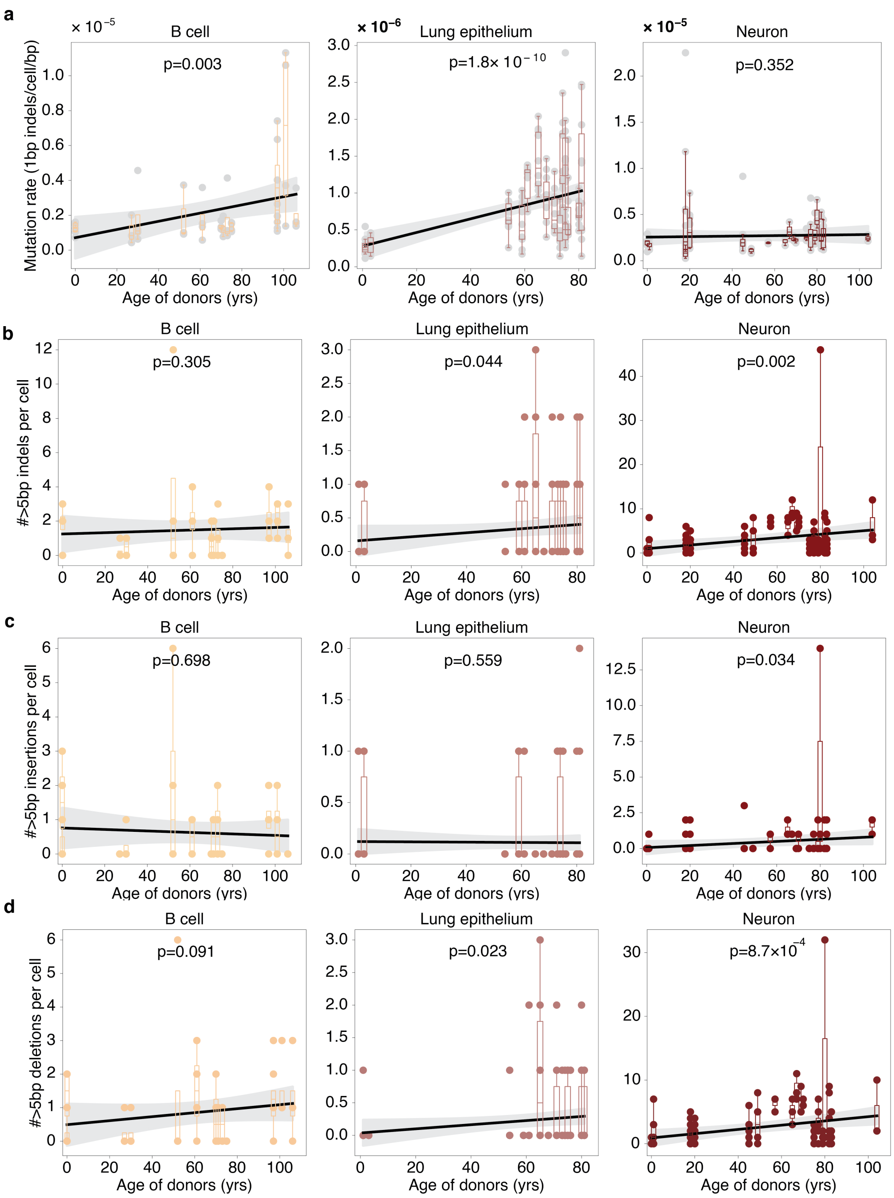

Supplementary Fig. 7 Mosaic STR indels across cell types. (a) 1-bp STR indels accumulate with age in B cells and lung epithelial cells. In contrast, the mutation rate remains relatively stable in non-dividing neurons across donors of different ages. **(b–d)** Relationship between age and the total burden per cell of STR indels >5 bp (b), STR insertions >5 bp (c), and STR deletions >5 bp (d) across three cell types. In all three cell types, the burden of STR deletions >5 bp shows a positive correlation with age. However, only in neurons does the burden of indels, insertions, and deletions consistently increase with age. Linear regression equations were derived using the least squares method. P-values were calculated using a one-tailed t-test to test whether the regression coefficient is significantly greater than zero. The shaded areas around each curve represent the 95% CIs, assuming the differences between predicted values and observed values follow a t-distribution.

bioRxiv preprint doi: https://doi.org/10.64898/2026.02.04.703915; this version posted April 5, 2026. The copyright holder has placed this preprint (which was not certified by peer review) in the Public Domain. It is no longer restricted by copyright. Anyone can legally share, reuse, remix, or adapt this material for any purpose without crediting the original authors.

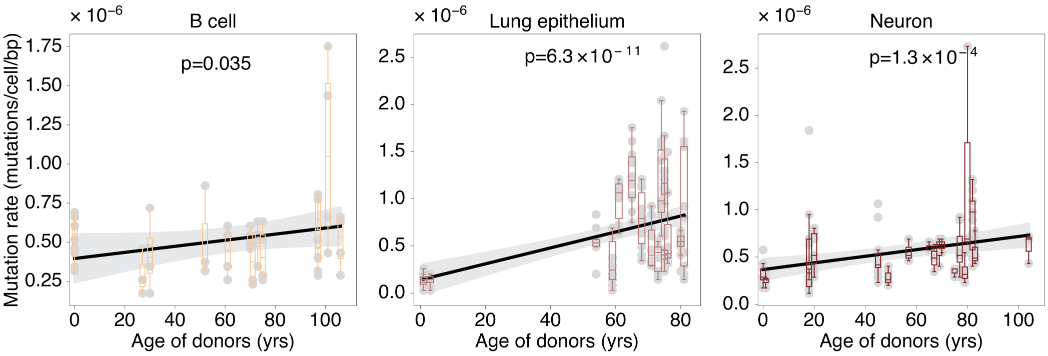

Supplementary Fig. 8 Mosaic STR SNVs across cell types. STR SNVs accumulate with age in all three cell types, as illustrated using the methodology described for Supplementary Figure 7.

bioRxiv preprint doi: https://doi.org/10.64898/2026.02.04.703915; this version posted April 5, 2026. The copyright holder has placed this preprint (which was not certified by peer review) in the Public Domain. It is no longer restricted by copyright. Anyone can legally share, reuse, remix, or adapt this material for any purpose without crediting the original authors.

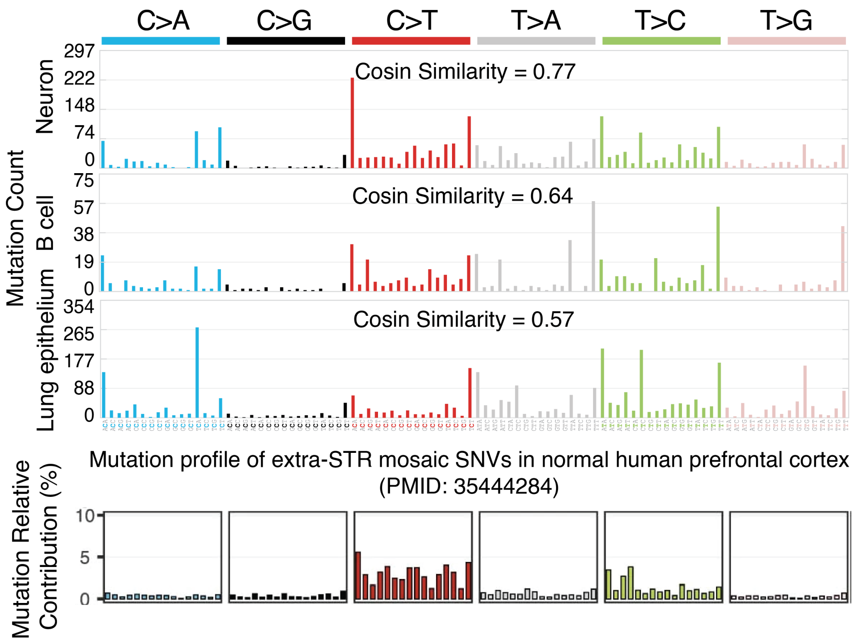

Supplementary Fig. 9 Mutational mechanism analysis of mosaic STR mutations. STR-region SNVs largely mirrored the established patterns of extra-STR mosaic SNVs in normal human prefrontal cortex from PMID 35444284. The mutation signature similarity was computed via cosine similarity.

bioRxiv preprint doi: https://doi.org/10.64898/2026.02.04.703915; this version posted April 5, 2026. The copyright holder has placed this preprint (which was not certified by peer review) in the Public Domain. It is no longer restricted by copyright. Anyone can legally share, reuse, remix, or adapt this material for any purpose without crediting the original authors.

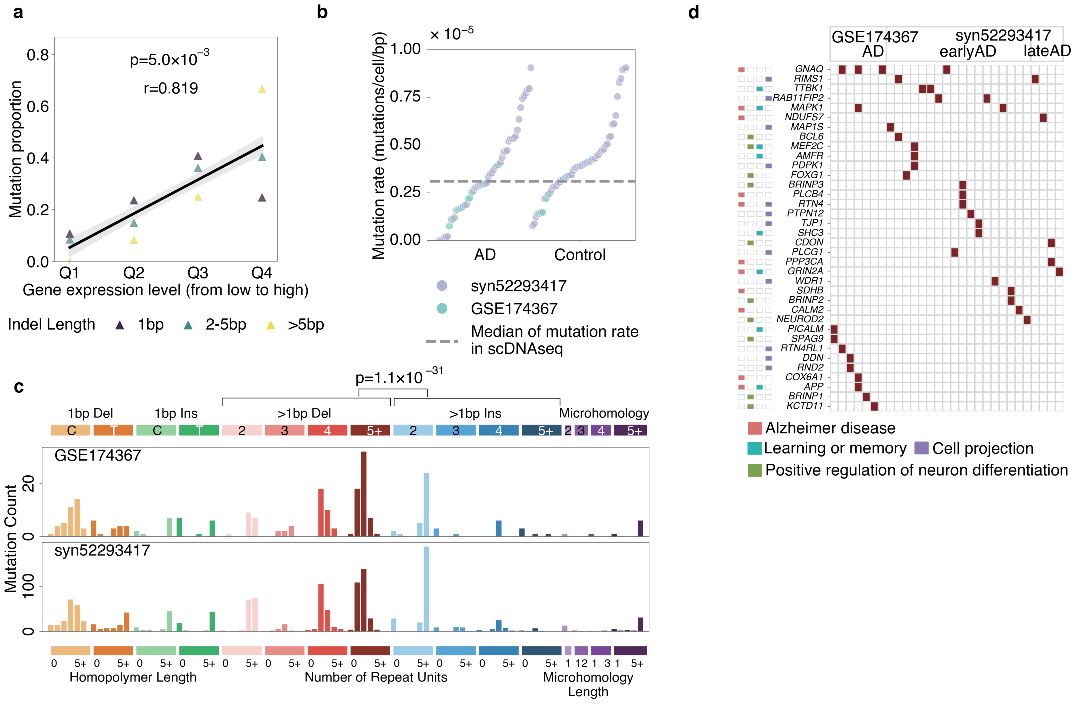

Supplementary Fig. 10 Regulatory mosaic STR indels in neurons. (a) Mosaic STR indels in neurons are enriched in TSSs and active enhancers of highly expressed genes. The number of variants is indicated beside each data point. Q1-Q4 represent expression level quantiles from low to high (Methods), and the brain gene expression data were obtained from ENCODE. Linear regression equations were derived using the least squares method. P-values were calculated using a one-tailed t-test to test whether the regression coefficient is significantly greater than zero. The shaded areas around each curve represent the 95% CIs, assuming the differences between predicted values and observed values follow a t-distribution. **(b)** snATAC-seq samples exhibit mutation rates highly consistent with those observed in scWGS data. **(c)** A significant excess of >1 bp deletions over >1 bp insertions was observed in snATAC-seq data. P-value was calculated using a one-tailed Binomial test. **(d)** Multiple key genes in AD pathways contained multi-hit STR indels in their active regulatory regions across different patients. “earlyAD” and “lateAD” indicate early-stage AD patients and late-stage AD patients.

bioRxiv preprint doi: https://doi.org/10.64898/2026.02.04.703915; this version posted April 5, 2026. The copyright holder has placed this preprint (which was not certified by peer review) in the Public Domain. It is no longer restricted by copyright. Anyone can legally share, reuse, remix, or adapt this material for any purpose without crediting the original authors.

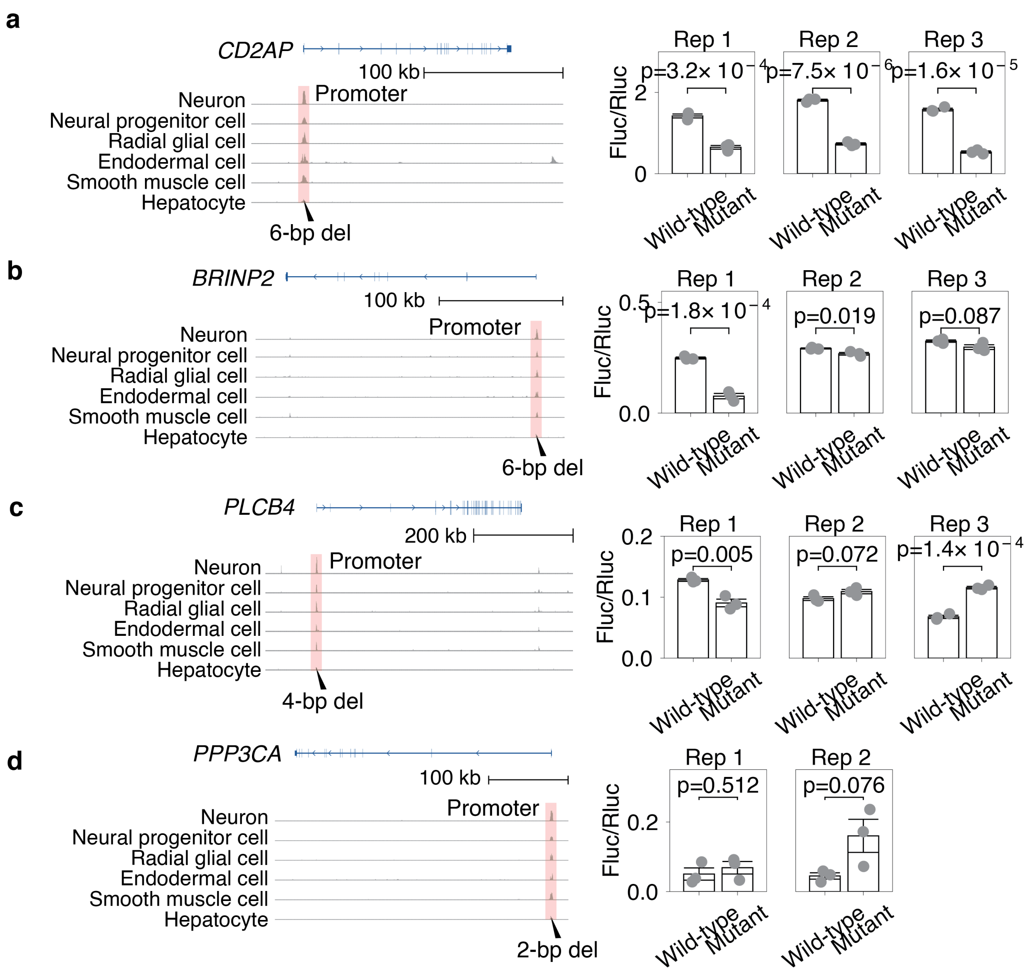

Supplementary Fig. 11 Luciferase assay results of somatic STR indels in regulatory regions. Normalized levels of H3K4me3 and H3K27ac—key histone modifications linked to promoter and enhancer activities across cell types were obtained from the ENCODE project. Pink regions indicate the promoter or enhancer regions of the gene, with indels highlighted by black triangles. Luciferase assays in HEK293T cells revealed that a 6-bp deletion in the TSS of *CD2AP* and a 6-bp deletion in the TSS of *BRINP2* significantly disrupted gene expression ($P < 0.05$, two-tailed t-test). Furthermore, a 4-bp deletion in the TSS of *PLCB4* and a 2-bp deletion in the TSS of *PPP3CA* enhanced gene expression with marginal significance ($P < 0.1$, two-tailed t-test). Error bars represent standard deviation (3 biological replicates).
